# Conventional and State-of-the-Art Detection Methods of Bovine Spongiform Encephalopathy (BSE)

**DOI:** 10.3390/ijms24087135

**Published:** 2023-04-12

**Authors:** Monika Olech

**Affiliations:** Department of Pathology, National Veterinary Research Institute, 24-100 Puławy, Poland; monika.olech@piwet.pulawy.pl

**Keywords:** BSE, bovine spongiform encephalopathy, diagnosis, PrP, prion

## Abstract

Bovine spongiform encephalopathy (BSE) is a fatal neurodegenerative disease that belongs to a group of diseases known as transmissible spongiform encephalopathies (TSEs). It is believed that the infectious agent responsible for prion diseases is abnormally folded prion protein (PrP^Sc^), which derives from a normal cellular protein (PrP^C^), which is a cell surface glycoprotein predominantly expressed in neurons. There are three different types of BSE, the classical BSE (C-type) strain and two atypical strains (H-type and L-type). BSE is primarily a disease of cattle; however, sheep and goats also can be infected with BSE strains and develop a disease clinically and pathogenically indistinguishable from scrapie. Therefore, TSE cases in cattle and small ruminants require discriminatory testing to determine whether the TSE is BSE or scrapie and to discriminate classical BSE from the atypical H- or L-type strains. Many methods have been developed for the detection of BSE and have been reported in numerous studies. Detection of BSE is mainly based on the identification of characteristic lesions or detection of the PrP^Sc^ in the brain, often by use of their partial proteinase K resistance properties. The objective of this paper was to summarize the currently available methods, highlight their diagnostic performance, and emphasize the advantages and drawbacks of the application of individual tests.

## 1. Introduction

Bovine spongiform encephalopathy (BSE) is a fatal neurodegenerative disease of cattle that belongs to a group of diseases known as transmissible spongiform encephalopathies (TSEs). The first huge epizootic of BSE was identified in Great Britain in 1986 and subsequently detected in cattle from many other countries worldwide. BSE has a long incubation period, between four to six years [1]. Clinical signs of the disease include behavioral abnormalities, hypersensitivity to touch and sound, gait abnormalities, weakness, and loss of body condition [2].

It is believed that the infectious agent responsible for prion diseases is abnormally folded prion protein (PrP^Sc^), which derives from a cellular protein (PrP^C^), which is a cell surface glycoprotein predominantly expressed in neurons. PrP^C^ is converted into the pathological form as a result of a conformational change, in which part of its α-helical structure is replaced by β-sheets [3,4]. The increase in the number of PrP^Sc^ conformers containing a high content of β-sheets motifs gives rise to hydrophobic PrP^Sc^ intracellular aggregates and form amyloid fibrils. PrP^Sc^ is toxic to cells of the central nervous system (CNS), and its formation is considered to be linked to the neurodegeneration of tissues within the CNS. Prions accumulate in high amounts in the brain, but low amounts of PrP^Sc^ are found in many tissues and biological fluids, even at the early stages of the presymptomatic period. PrP^Sc^ is partially proteinase kinase (PK)-resistant, resulting in the formation of an N-terminally truncated fragment known as PrP^res^ (res meaning resistant). This unique feature of this protein has been used to develop most of the testing methods currently used to detect of BSE. The clinical manifestations of the disease, although characteristic, are insufficient for a definitive diagnosis. The immune system is unable to produce a specific response to PrP^Sc^ because the protein is not recognized as foreign. Therefore, immunological tests that are based on the detection of an immune response cannot be used for the diagnosis of BSE. The disease is diagnosed mainly post-mortem by detecting PrP^Sc^, which is, so far, the only validated marker for BSE.

Three types of BSE have been detected (Table 1). Classical (C-BSE) BSE is the most common type of BSE, which was responsible for the appearance of BSE in Great Britain in 1986. Two additional types, atypical BSE, were discovered in 2004 in Italy and France [5,6]. They were defined as L (low) type and H (high) type, according to the lower and higher positions of the unglycosylated PrP^res^ band in the Western blot, respectively, compared to the position of the band in the classical form of BSE. The L type is also termed bovine amyloidotic spongiform encephalopathy (BASE) due to the presence of PrP-positive amyloid plaques in the brain not observed in classical BSE. L- and H-BSE are less common but have been reported in several European countries as well as the United States of America, Canada, and Japan [7]. Classical BSE was propagated through feeding meat and bone meal originating from infected cattle. Atypical BSE forms appear to occur spontaneously in older animals and without a causal link to the feed-borne C-BSE epidemic. Most cases of atypical BSE were identified in fallen stock, and none was reported as clinically suspected. H-type and L-type BSE diseases are characterized by low head carriage, dullness, lack of nervousness or inactivity, suggesting that the clinical presentation is unlike C-BSE. However, the link between classical and atypical forms of BSE is still unknown. The use of bovine material derived from animals infected by the atypical form of BSE in feed and food production may therefore pose a risk for humans. The use of rapid tests that can reliably detect such cases is, therefore, crucial in the frame of the protection of the consumer from accidental exposure to the BSE agent [8].

BSE is the only known zoonotic animal TSE, causing a variant of Creutzfeldt–Jakob Disease (vCJD) in humans. The zoonotic potential of the atypical forms is unknown, but some laboratory transmission studies indicated the zoonotic potential of the L-BSE form, which appears to be even higher than that of the C-BSE form [9,10,11,12,13]. Additionally, cross-species transmission of C-BSE has been observed in exotic ungulates, cats, and goats in field conditions [14]. BSE can also be experimentally transmissible to sheep, goats, and pigs [15]. There are concerns that the BSE agent may also circulate in the sheep and goat populations, posing a possible secondary risk to humans [14,15,16]. Goats and sheep orally inoculated with BSE develop a disease clinically and pathogenically indistinguishable from scrapie. Therefore, in cases of TSE in small ruminants, differential testing is required to determine whether the TSE is scrapie or BSE.

Many BSE detection methods have been developed and have been reported in numerous studies. The purpose of this paper was to provide a summary of currently available BSE diagnostic methods, highlight their diagnostic performance, and emphasize the advantages and disadvantages of the using of each test.

## 2. Sampling

For diagnosis of BSE, the whole brain should be sampled. Early accumulation of PrP^Sc^ and vacuolar changes in the medulla oblongata at the level of the obex make this area of the brain reliable material for diagnosis of BSE. Samples from the obex are conveniently obtained by a long spoon-shaped metal or disposable instrument with cutting edges through the foramen magnum. PrP^Sc^ can be detected in unfixed brain extracts by immunoblotting and other enzyme immunoassay methods and can be detected in a fixed brain by immunohistochemistry (IHC). For neuropathological examination, one block 0.5–1 cm in width taken from the medulla oblongata at the level of the obex must be collected. Formalin-fixed and paraffin-embedded samples are used for spongiform changes and PrP^Sc^ detection by histological and immunohistochemical methods, respectively. For immunodetection of PrP^Sc^, fresh material as a hemisection of the medulla at the level of the obex or complete coronal section (immediately rostral or caudal to the above area) should be taken. The sampling of the brain tissue for use in rapid tests should be performed according to the manufacturer’s protocols since these vary from method to method. These commercially available tests can be performed within 24 h and allow screening of large numbers of samples. The preferred samples should be within 1.0 cm rostral or caudal to the obex in order to include key target sites of PrP^Sc^ accumulation. When rapid tests are used as the primary method, it is important to make material available for further confirmatory tests. Imprecise hemisectioning could result in the complete loss of a target area for confirmatory testing [17].

## 3. Histopathological Examination

**Figure 1:**
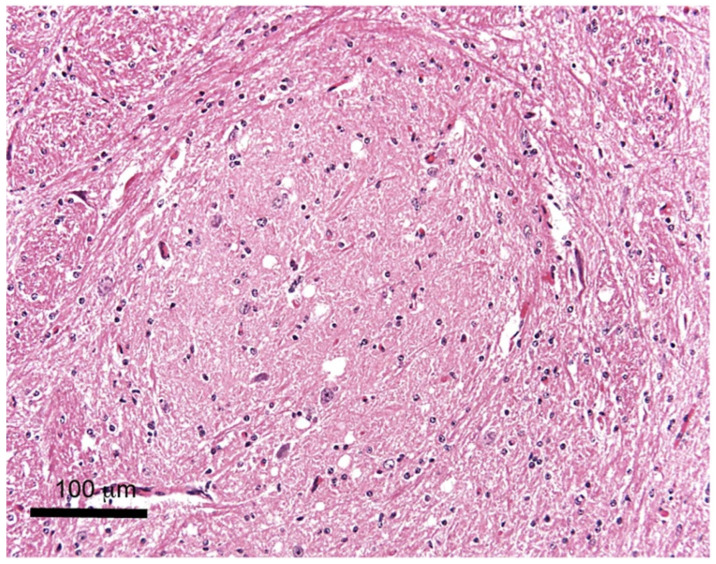
Hematoxylin and eosin staining, the nucleus of the solitary tract of H-type BSE: the presence of spongiosis in the neuropil (100 μm). Reprinted from Ref. [18].

Histopathology was the first method for TSE diagnosis that allowed the detection of TSE lesions in the tissue of the central nervous system. The area of the brain routinely processed is the medulla at the level of the obex and should be fixed in 4% formaldehyde solution (10% formal saline or 10% normal buffered formalin) for a minimum of 3–5 days. Formaldehyde cross-links with protein, stopping autolysis and bacterial degradation. Fixed tissues should then be treated with 98% formic acid for one hour to decrease prion infectivity and then washed in water. In the next step, tissue is embedded in paraffin blocks and cut at 5 µm thickness sections, then stained with hematoxylin and eosin (HE). HE-stained sections are analyzed under a light microscope to determine the severity and distribution of vacuolation. Vacuolation of the gray matter neuropil (spongiform change) and/or vacuolation of neurons that are bilaterally distributed are the most common histological changes that can be observed in the central nervous system of BSE cases (Figure 1). 

**Figure 2:**
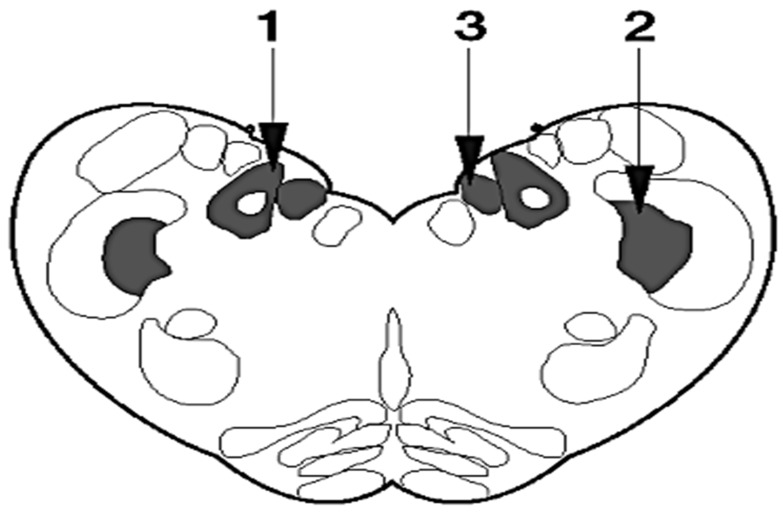
Cross-section of the bovine obex identifying the target sites for BSE diagnosis: (1) nucleus of the solitary tract; (2) nucleus of the trigeminal nerve; (3) dorsal motor nucleus of the vagus nerve. Reprinted from Ref. [17].

The highest accumulations of these vacuolar changes are located in the specific areas of the brainstem at the level of the obex, in the solitary tract nucleus and spinal tract nucleus of the trigeminal nerve, and in the dorsal nucleus of the vagus nerve [19]. Therefore these areas are used for histological diagnosis of BSE (Figure 2). Intraneuronal vacuolations are also incidentally observed in BSE. Thus, the presence of vacuolated neurons alone, in the absence of neuropil vacuolation, is not a confirmation of BSE [20]. Thus, histopathology alone is no longer the diagnostic method of choice for the examination of suspicious animals or the screening of healthy populations. 

**Figure 3:**
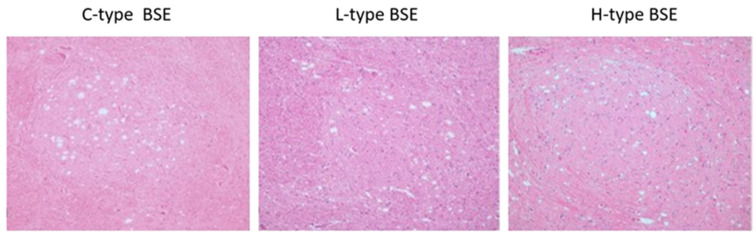
Hematoxylin and eosin (H & E)-stained sections of the solitary tract nucleus of classical and atypical BSE cases. Reprinted from Ref. [21].

Examination of the medulla oblongata at the level of the obex does not allow a comprehensive phenotypic characterization of TSEs, since spongiform lesions are similar in all animal TSEs. The neuroanatomical distribution of vacuolation and its appearance are usually not distinguishable also between H-type, L-type, and C-type cases of BSE (Figure 3) [21,22]. Only in the first L-BSE cases, were spongiform changes not consistently found in the brainstem, at the level of the obex. Therefore, this finding may call into question the detectability of the disease in weak cases of L-type BSE since histopathology may not yield positive results [23].

A numerical lesion profile for a TSE is produced according to the vacuolation severity in selected areas of the brain. Histopathology is known to have some limitations. It was shown that 5% of clinically suspect BSE cases could not be confirmed histopathologically. It was also reported that some BSE cases display typical clinical signs with minimal or undetectable vacuolar lesions [24]. Imprecise hemisectioning could result in the complete loss of a target area for confirmatory testing. Furthermore, histopathology requires good sample preservation. Therefore, the brain should be extracted as soon as possible after animal’s death by standard methods. However, in some cases, especially in the summer due to the high temperatures and delays in post-mortem examination, the brains undergo severe autolytic degradation that makes samples unsuitable for histological examination. Debeer et al. demonstrated that freezing before fixation, compared to freshly-fixed material, caused pronounced histological damage. Irrespective of autolysis, low-quality fixation and paraffin-embedding can give rise to artefactual vacuoles leading to limited interpretation [25]. Therefore, to minimize the effects of subjective examination, the data should be obtained by two experienced pathologists and with a set of reference controls. Although histopathology is very specific because it allows direct observation of the signs of the disease and has lower sensitivity than other methods, it has been almost completely superseded by them. The sensitivity of microscopic observation can be increased by immunohistochemical methods that use PrP-specific antibodies for the detection of PrP^Sc^ amyloid deposits.

## 4. Immunohistochemistry (IHC)

IHC examination is conducted on formalin-fixed paraffin-embedded brain-derived tissues to highlight the presence of PrP^Sc^ accumulation. Detection of PrP^Sc^ relies on its protease resistance and detection withanti-PrPantibodies. The main difficulty with antibodies for BSE is that these cannot specifically discriminate PrP^C^ from PrP^Sc^. Therefore, multiple pretreatments (a combination of chemical, enzymatic, and heating treatments) are necessary to suppress the recognition of normal cellular prion protein PrP^C^ and enhance antigen retrieval of disease-associated prion protein (PrP^Sc^) antigens after fixation. Various types of pretreatments in different possible combinations were described for PrP immunohistochemistry. Most common pretreatments consist of a 98–100% formic acid bath followed by hydrated autoclaving at 121 °C, and then digestion with proteinase K. Enzymatic digestion with proteinase K, formic acid, and autoclaving destroy the unstable PrP^C^ but preserve the detection of PrP^Sc^. An alternative way is chemical pretreatment. The merit of chemical pretreatments using KMnO_4_ for PrP immunohistochemistry seems to be the complete destruction of PrP^C^ by strong oxidation [26]. However, a combination of strong oxidation using KMnO_4_ and protein denaturation using N-lauroylsarcosine can frequently alter tissue adherence [26]. Okada et al. described a chemical pretreatment step using the combination of a high concentration of NaOH and a high temperature enhanced PrP^Sc^ immunolabeling in comparison with the chemical method using KMnO_4_. The chemical pretreatment method is easy to use and, compared to autoclaving, reduces the sample processing time and equipment cos, since only a cheap water bath is required. Furthermore, Okada et al. revealed that immunolabeled PrP^Sc^ pretreated with autoclaving generated more nonspecific background staining compared to chemically pretreated PrP^Sc^ [27]. It has been speculated that autoclaving alters the three-dimensional (3D) structure of the formalin-modified antigen, so the application of heat in the improved chemical method may restore the native conformation of PrP [28]. 

In IHC, after pretreatment and two blocking steps (endogenous peroxidase activity is blocked in 3% H_2_O_2_ (hydrogen peroxide) in methanol, and nonspecific tissue antigens are blocked by incubation with horse blocking serum), incubation with a primary monoclonal antibody directed to prion protein is carried out. After rinsing, a secondary antibody is applied. PrP^Sc^ immunoreactivity is visualized using chromogen and then counterstained with Meyer’s hematoxylin, rehydrated in ethyl alcohol and xylene, and coverslipped. Positive immunoreactivity occured from light to dark brown and corresponded to the distribution of vacuolar changes in the BSE brain. PrP^Sc^ can also be detected using the immunofluorescence technique using a laser scanning confocal microscope [29]. Electron microscopy (EM) has unquestionably increased the magnification and resolution of pathology pictures, and despite the fact that this technique is not used routinely used for diagnosis purposes due to the expertise required and the associated cost, it remains a very powerful research tool, especially in combination with IHC.

Ten morphological forms of PrP^Sc^ deposition were identified: granular, intraneuronal, perineuronal, linear, intraglial, stellate, fine punctate, perivascular, coalescent, and plaque-like [30,31,32]. In the intraneuronal form, which is mainly observed in the dorsal motor nucleus of the vagus nerve, fine-to-coarse granular PrP^Sc^ deposits are scattered in the cytoplasm of neurons. The intraglial form is characterized by fine punctate PrP^Sc^ immunoreactivity found adjacent to the glial nuclei. In the granular type, multiple small PrP^Sc^ granules are widely distributed in the neuropil of the gray matter nuclei. The perineuronal type comprises thin deposits of PrP^Sc^ around single neuronal neurites and perikarya, while the linear type is characterized by thread-like PrP^Sc^ deposits, particularly seen at the level of the reticular formation of the brainstem. The coalescing type appears to result from the aggregation of granular PrP^Sc^ deposits into large amorphous or mesh-like masses, while in the stellate form (also named glial), PrP^Sc^ deposits branch out from the glial-type nucleus, forming a star-shaped appearance observed in the cerebral lamina and central grey matter, and within the medial pontine nuclei in the thalamus, cerebral cortex, and obex. In the perivascular form, PrP^Sc^ deposits are located around the blood vessels in the white matter. In the fine punctate form, numerous small PrP^Sc^ granules are observed in the neuropil, while in the plaques-like form, radiate, fibrillary, and relatively large Pr^Sc^ accumulations are mainly distributed around blood vessels of a different caliber [33]. Extensive small PrP-positive amyloid plaques are the hallmark characteristic of the L-type of BSE (BASE). 

Numerous studies have described the PrP^Sc^ profile for all BSE types, but a comparison of the obtained results is difficult due to the various factors, including the different brain regions used for the analyses, and differences in the methodology, especially in the antibodies used and PrP^Sc^ types. Thus, the obtained findings often gave contradictory results [20,22,34,35]. Nevertheless, it was found that at the level of the obex, there are no evident differences in the abundance and distribution of PrP^Sc^ immunolabelling between C-type BSE and atypical BSE. Only Konold et al. revealed subtle phenotype-specific differences in this area, specifically labeling in white matter tracts in the H-type BSE cases and minor immunolabelling aggregates in the entirereticular formation in L-type BSE cases [21]. Greater qualitative discrimination was possible by testing more rostral regions of the brain. The cerebellum has proven to be a more reliable sample for discriminating BSE types. L-type BSE cases showed a very homogenous diffuse PrP^Sc^ immunolabelling in the molecular and granular layers of the cerebellum and perineuronal depositions in the granular layer. In H-type BSE, the immunolabelling in the molecular and granular layers of the cerebellum was minimal, but strong intramicroglial labeling throughout the white matter of the spinal cord and the cerebellum was observed. The most characteristic feature in C-type BSE cases was the variability in the reaction patterns [21,22]. 

IHC can also be used for discriminating BSE and scrapie in sheep. The exact basis for differentiation is the location of the N-terminal cleavage site for PK digestion of PrP^Sc^ between scrapie and BSE. It was revealed that the N-terminal amino acid sequence WGQGGSH remains intact only in sheep with scrapie and therefore can be identified by antibody P4 specific for this N-terminal domain. This approach proposes that BSE and scrapie can be recognized by the distribution of immunolabelling by using antibodies directed to both the C-terminal and N-terminal of the PrP^Sc^ protein. In the case of BSE, a significant reduction in the intracellular PrP^Sc^ signal occurs when N-terminal antibodies, such as P4, are used. Furthermore, the defining of morphological types of PrP^Sc^, the levels of PrP^Sc^ accumulation, as well as cell-type associations, across various neuroanatomical sites, also helps the distinguishing of ovine BSE from scrapie. This technique has been referred PrP^Sc^ profiling and depends on the antibody used. However, the main drawback of this method is the subjective interpretation of PrP^Sc^ staining [16,36]. 

Some results suggest that the lymphoreticular system (LRS) can be used to discriminate C-type-BSE- and scrapie-infected sheep using the above-mentioned dual antibody staining. It is possible because C-type-BSE-infected sheep show infectivity of lymphoid tissues similar to scrapie, where the LRS is mainly engaged in the transport and the spread of the agent [37,38]. However, Matsuura et al. reported no specific PrP^Sc^ staining in the lymphoid tissue of sheep inoculated with cattle L-BSE, suggesting that this strain has distinct tissue tropism in sheep [39]. The pathogenesis of BSE in cattle varies significantly from that of TSE in sheep. It has been shown that, in contrast to most other animal species, in cattle, the BSE is amplified almost entirely in the central and peripheral nervous systems. Therefore, the LRS in cattle cannot be used for the discrimination of BSE types since PrP^Sc^ is not detected in the peripheral lymphoid tissues in atypical and classical cases of BSE [21,29,40,41]. 

The efficiency of the IHC method depends on sample preparation and the antibodies (Abs) used. Many specific antibodies against PrP have been produced (Table 2). However, since PrP^C^ and PrP^Sc^ share a primary structure, most of the anti-PrP Abs can bind to both isoforms, which are referred to as pan-PrP Abs. R145 is a rat monoclonal antibody (mAb) to PrP. Its synthetic peptide corresponds to the sequence YQRESQAYYQRGA (221–233) of bovine PrP. Most other monoclonal antibodies (2G11, F99, F89, 12F10, L42, 6H4, KG9, SAF84, 2A11) are produced in mice. Care should be taken in selecting the correct biotinylated secondary antibody. Anti-mouse antibodies should be used for antibodies raised in mice, and anti-rat for R145. A wide spectrum of anti-PrP monoclonal and polyclonal antibodies can be used, and an increasing number are now commercially available. These antibodies recognize epitopes situated at the N-terminal end of the PrP molecule (BG4, SAF32, B103, P4, 8G8, 521, 505, 12B2), the C-terminal end of the PrP (T4, F99/97.6.1, R145, 6H10), and the core region (T1, 12F54, F89/160.1.5, 44B1, SAF84, 43C5, F89, 6H4, L42, 2G11) [16]. Several factors are involved in the sensitive detection of PrP^Sc^ using monoclonal and polyclonal antibodies. These factors include the configuration of the proteins and, thus, the epitope orientation and presentation, as well as the precise proteinase cleavage sites, which are distinct between different types of the prion proteins [20]. The reactivity of mAb to prion protein also depends on the analytical method, as different antigen treatments in different methods resulted in the exposition of certain epitopes in a conformation-dependent manner and affected the affinity of antibodies and antigens. For example, the B4 antibody reacted with both PrP^C^ and PrP^Sc^ in the IHC test, but it showed a faint reaction with PrP^C^ in the Western blot test [42].

IHC is believed to be a highly sensitive and specific tool for PrP^Sc^ detection and is currently the most widely used confirmatory tool for both passive and active surveillance. PrP^Sc^ immunolabeling is particularly useful when there is no vacuolization or where vacuolization is minimal. IHC is a more effective tool than conventional histology but is time-consuming, and includes subjective interpretation of PrP^Sc^ staining patterns; thus, it is not suitable for large-scale typing. Furthermore, IHC is usually performed on fixed materials. However, Chaplin et al. showed that the standard IHC method was able to stain PrP^Sc^ in autolyzed samples without the need for modification of the staining techniques [59]. Furthermore, Debeer et al. revealed that IHC could be performed onfixed and frozen brain samples with a high level of sensitivity without generating unspecific background staining [27,60]. Fixed-frozen brain samples retained their integrity during the histological preparation process (formic acid incubation, autoclaving, and proteinase K digestion) despite the anticipated cell membrane disruption. Similarly, very satisfactory adhesion of the brain sections to glass was found. Nevertheless, freezing prior to fixation, compared to freshly-fixed material, caused evident histological damage, such as scar-like tears [60]. Furthermore, immunocytochemistry is a useful tool for the diagnosis of samples with an advanced degree of autolysis, particularly those in a liquid state, which presents a difficulty for diagnosis. As was demonstrated by Monleon et al., such autolyzed material was resistant to all pretreatments for antigen unmasking and was preserved on the glass slides. Background immunostaining was observed in such samples, but was always distinguished from the characteristic granular-positive deposits [61]. Sarasa et al. also revealed that immunocytochemistry is a suitable technique capable of confirming BSE-positive samples with advanced autolysis when rapid tests, IHC, and Western blot gave non-conclusive results [62].

## 5. The Paraffin-Embedded Tissue (PET) Blot and Histoblot

Schulz-Schaeffer et al. developed a sensitive technique for detecting PrP^Sc^ in formalin-fixed and paraffin-embedded tissues, called the paraffin-embedded tissue blot (PET blot) [63]. PET blot is a modified version of standard IHC and thus is easy to perform as a routine procedure at the same time as conventional immunohistochemistry. In this method, 5 µm sections of paraffin-embedded tissue are cut on a microtome and placed on prewetted nitrocellulose or PVDF-immobilon membranes supported on glass slides. Then the membrane is dewaxed, rehydrated, and dried. Next, digestion with proteinase K is performed. The PET blot, similar to the Western blot method, requires tissue pretreatment with high concentrations of proteinase K that ensures complete digestion of all PrP^C^ proteins, whereas PrP^Sc^ is only partially degraded to PrP^res^. As a result, only PrP^Sc^ is detected by this method. With this step, the membrane-attached proteins are fixed to the membrane. Immunodetection is performed after incubation in a blocking solution to block nonspecific protein binding sites on the membrane. Then incubation with the primary antibody followed by incubation with species-specific secondary antibodies is performed. After intensive washing, the membranes are adjusted to alkaline pH by incubation in the alkaline phosphatase reaction buffer containing 100 mM Tris-HCl [pH 9.5], 100 mM NaCl and 50 mM MgCl_2_. Finally, 5-bromo-4-chloro-3-indolyl phosphate/nitroblue tetrazolium (NBT/BCIP) is used to visualize the reaction product. Blots are evaluated using a dissecting microscope [63].

It has been shown that the PET blot is an extremely highly specific and sensitive technique for PrP^Sc^ detection. It is more sensitive than immunohistochemistry, histoblot, and Western blot methods. Furthermore, PET blot allows the detection of PrP^Sc^ during the incubation phase long before the occurrence of clinical disease when the level of PrP^Sc^ is very low. Investigation with intracerebrally infected mice demonstrated that the PET blot was able to detect PrP^Sc^ in the brain 30 days after infection and 145 days before the onset of clinical signs [63]. 

The use of fixed and paraffin-embedded tissues prior to proteinase kinase digestion offered better preservation of the samples in comparison to the histoblot performed on unfixed frozen samples. In the histoblot technique, frozen tissue sections are mounted on a nitrocellulose membrane and then treated and developed in the same manner as Western blot. PET blot is very useful when the only material available from samples is in the form of paraffin-embedded tissues, as is often the case in archival studies [64]. However, care should be taken when selecting tissue for PET blot, as tissues treated with formic acid for decontamination prior to tissue processing and embedding in wax showed a significantly reduced amount of immunolabeling detected with P4 [65].

Conventional immunohistochemistry has a better microscopic resolution than PET blot and histoblot at the cellular and subcellular levels. However, PET blot allows greater anatomical resolution compared to histoblot. It may be of particular interest in biopsy diagnosis and useful in the detection of very small amounts of PrP^Sc^ in tissues. However, limited detection of PrP^Sc^ in lymphoid tissues of animals with BSE does not offer the possibility of using these tissues for preclinical diagnosis using biopsy. These tissues can be used to distinguish scrapie- and BSE- infected sheep [37,38]. 

Even if PET blot lacks a high cellular precision, which only IHC offers, it can be used to discriminate scrapie and ovine BSE cases using specific antibodies. Webb and other authors revealed that using the mAb R145, the signal was strong in every case of experimental bovine BSE or scrapie, but the amount of immunostaining with P4 was significantly decreased or absent in experimental ovine BSE cases [65]. Furthermore, PET blot offers scope to extend discriminatory testing in cases where the IHC target areas are not present since the loss of immunolabeling with P4 is not limited to these areas only, as is in the case of IHC. It was shown that the differentiation was easier with PET blot than with immunohistochemistry, with precise observations possible at the macroscopic level. The discrimination by PET could be made with greater certainty than by IHC, where the differences were very subtle. The PET blot method appears to be a rapid and precise method for studying distribution of PrP^res^ in the infected brain; however, the presence of PrP^res^ is not linked to the presence of vacuolar lesions. PET blot cannot be used alone for the identification of PrP^Sc^, as in some cases PrP^res^ is present at a minimal or undetectable level [64]. PET blot is not a rapid method for testing large numbers of animals, such as in a slaughterhouse. However, with superior sensitivity and excellent specificity, it provides an excellent tool for the verification of the test results. 

In 2005, Polak et al. developed the dot blot method for the diagnosis of BSE. This method is simple, equipment requirements are minimal, and testing time is short. In this assay, homogenized brainstem samples after digestion with proteinase K are applied on a 96-well plate with PVDF membrane at the bottom and developed in the same way as Western blot. Thus this method is a good candidate for screening purposes. This test was able to detect all positive cases and had higher sensitivity than the Prionics^®^-Check Western rapid test [66].

## 6. Western Blot Methods

Western blot (WB) is the most common technique used for the diagnosis of prion diseases. A variety of Western blot methods have been developed. Some are used as screening tests, while others are used to confirm the suspected cases identified through active and passive surveillance. WB methods are very versatile since they can be applied to fresh (unfixed), frozen, and autolytic tissues. The techniques are based on the immunodetection of the PrP^Sc^. Therefore, samples are subjected to PK digestion, which fully digests the normal PrP^C^, while proteolysis of PrP^Sc^ is restricted to the unstructured N-terminus leading to the protease-resistant product PrP^res^ (27–30 kDa), which is smaller than the original size of PrP^Sc^ (32–35 kDa). Anti-prion antibodies are then used to detect the intact PrP^res^. 

Western blotting requires preparation of brainstem homogenates and digestion of the samples with proteinase K. After ultracentrifugation, the pellet is dissolved in a Laemmli buffer, and the sample, after denaturation, is loaded onto SDS-polyacrylamide gels (SDS-PAGE). Denaturation allows the binding of the protein with the antibody-recognized PrP^Sc^. After separation, the denatured proteins are transported onto a polyvinylidene difluoride (PVDF) membrane and detected with an enzyme-labeled antibody. The signal is visualized by a chemiluminescence system using Hyperfilm ECL sheets. Initially, ‘OIE Western immunoblot’ (also known as the ‘OIE-SAF Western blot’) protocols were lengthy procedures with several ultracentrifugation steps needed to concentrate the PrP^Sc^, which required a large amount (2–4 g) of starting material. Current Western blot methods use smaller amounts of brain material (up to 0.5 g) and, due to the use of appropriate homogenization, buffers do not need the ultracentrifugation steps; hence these methods are less time-consuming [20,67]. 

The OIE and national reference laboratories have established WB methods to discriminate H- and L-type BSE from classical BSE, and a detailed protocol including diagnostic criteria is available online at TSEglobalNet [68]. In positive cases, Western blot shows the presence of PrP^res^ characterized by an electrophoretic pattern consisting of three bands that correspond to the diglycosylated (27–30 kDa), mono-glycosylated (23–25 kDa), and unglycosylated (18–21 kDa) forms. Each glycoform has a characteristic molecular weight and amount depending on the BSE type therefore glycoform ratios and molecular weights are used to distinguish the type of BSE. Classical BSE has a glycoform ratio of 60% di-glycosylated, 28% mono-glycosylated, and 12% unglycosylated with bands at around 28, 22, and 18 kDa, respectively [69,70,71]. The H-type BSE PrP^res^ is slightly larger, migrated more slowly, and is detected at a higher position than classical BSE. The glycoform ratios are very similar to classical BSE. H-type BSE is characterized by a considerably larger molecular size of the unglycosylated PrP^Sc^ form [6,70]. The higher molecular mass of the unglycosylated molecules results from the presence of an additional ~10 kDa fragment, which is detectable only with 94B4 and SAF84 antibodies binding to the C-terminal region from position 154–236 [72]. L-type BSE PrP^res^ is smaller, migrates faster, and is detected in a lower position than classical BSE during electrophoresis [73]. L-type has only a slightly smaller molecular size of the unglycosylated PrP^Sc^ form and differences in molecular weight of this form between L-BSE and C-BSE PrP^Sc^ are just 0.3 kDa, so they are not suitable for routine prion typing [69,70,71]. H- and L-type BSE cases had a higher proportion of the monoglycosylated band and a lower proportion of the diglycosylated band than C-type BSE. Notably, L-type BSE showed an equal ratio between di-glycosylated and mono-glycosylated isoforms, which is a characteristic feature of L-type BSE [35]. No PrP signals are present in the negative samples since the PrP^C^ is completely digested by proteinase K. 

**Figure 4:**
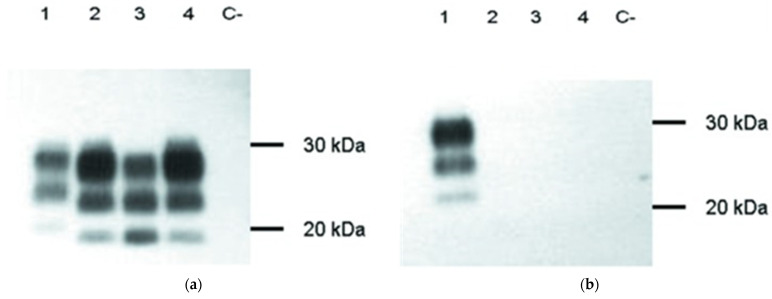
Western blot analysis. H-type BSE (1), C-type BSE (2), L-type BSE (3), and C-type BSE (4) samples and healthy bovine used as negative control (C-). mAbs 6H4 (**a**) and P4 (**b**) were used, respectively. Adapted from Ref. [35].

H-type cases were also characterized by strong labeling with P4 and 6H4 monoclonal antibodies. Both of these antibodies are directed to the N-terminal epitope that is present after proteinase K cleavage only in H-type BSE, but not in C- and L-type BSE-derived PrP^Sc^. When the 6H4 mAb, which is directed against the protease-resistant core of the PrP, was used, all BSE types (C, L, and H) gave positive results. In contrast, when P4, which is directed against the N-terminus of the protease-resistant core, was used, only H-type BSE samples were recognized (Figure 4) [35,74]. The observed differences resulted from different PK cleavage sites for all three BSE types, as described by Jacobs et al. [69]. 

Digestion with two different conditions, stringent (pH 8, 500 µg/mL PK) and mild (pH 6.5, 50 µg/mL PK), also allows the discrimination of BSE types [69]. C-type BSE is remarkably resistant to degradation by protease digestion under stringent conditions (pH 8, 500 µg/mL PK). In contrast, L- and H-type atypical BSE prions are generally less resistant and can be degraded by digestion under rigorous conditions with proteinase K. Therefore, under rigorous conditions, the H- and L-type PrP^res^ signals decreased more rapidly, while the C-type PrP^res^ signal remained almost constant [75,76]. The PK susceptibility ratio is obtained by comparing the optical density of the signal strength of the PrP^res^ bands arising from mild and strong digestion. According to Jacobs and others [69], the susceptibility ratio for C-BSE is more than 50%, while for L- and H-BSE cases it is less than 20%. However, sometimes differences were very subtle and not easily detected by Western blot.

**Figure 5:**
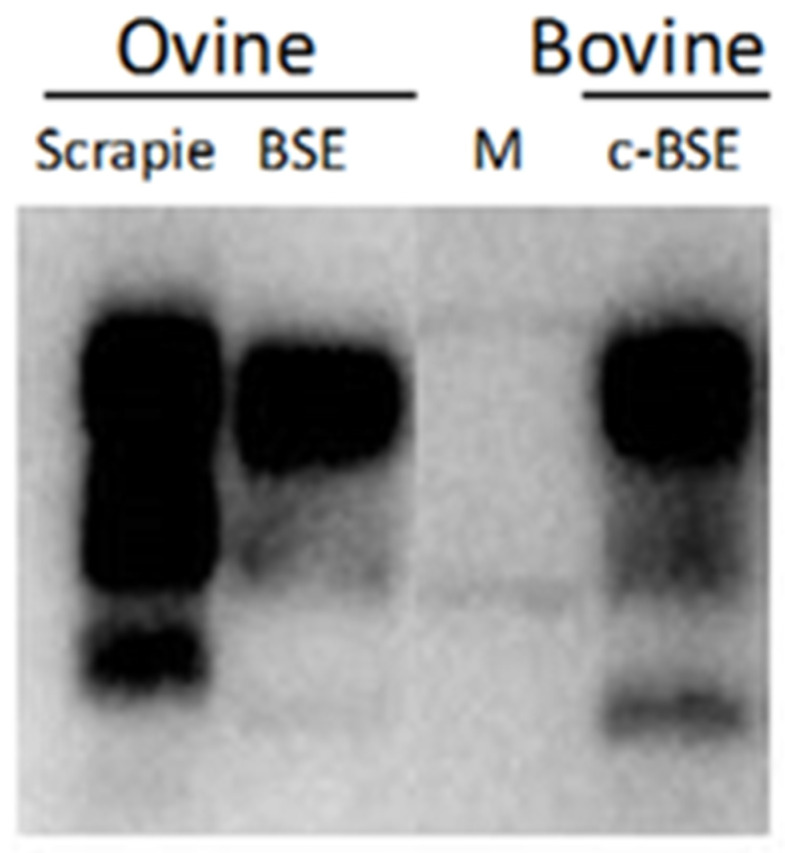
Western blot detection of PrP^res^ from proteinase K-treated and ultracentrifuged brain homogenates, using a core antibody. Ovine scrapie, ovine BSE, and bovine C-type BSE are shown. The lower molecular weight of the unglycosylated protein band in ovine or bovine BSE compared to scrapie is evident. M-molecular mass markers 20 and 30 kDa. Reprinted from Ref. [79].

Promising results for discriminating sheep scrapie and sheep BSE have been obtained using Western blot. Some studies have identified a larger molecular mass of the unglycosylated band of PrP^res^ in sheep with natural scrapie than in experimentally BSE-infected sheep (Figure 5). An unglycosylated band of PrP^res^ in BSE sheep is approximately 1.6 kDa smaller than in sheep scrapie [6,21,53,77,78,79,80,81]. Unfortunately, the unglycosylated fraction represents only 5–20% of total PrP^res^, and the unglycosylated band can be very faint or invisible, making this approach less attractive for routine diagnosis. Indeed, the unglycosylated band became more intense after the N-glycosidase F (PNGaseF) treatment when most of the di- and mono-glycosylated PrP^res^ disappeared. Although such deglycosylation is often incomplete, in routine diagnostic experiments, the unglycosylated band becomes sufficiently dense to distinguish scrapie-infected sheep from those with experimental BSE [16,53]. 

However, the glycoprofiles and electrophoretic mobility of the unglycosylated fraction of PrP^res^ from the brains of sheep infected with the scrapie-like strain CH1641 were indistinguishable from those of sheep experimentally infected with BSE and different from sheep with natural scrapie. In such cases, only immunohistochemical methods applied to PrP^Sc^ examination could clearly discriminate ovine BSE and experimental CH1641 infection [16]. 

Differences in the molecular mass of PrP^res^ between ovine BSE and scrapie can be as little as 0.4 kDa, and such subtle differences cannot be easily detected using Western blot. Therefore, differential antibody binding is another aspect that can be used for discrimination using the Western blot method. It has been suggested that PrP^res^ from animals infected by BSE are more susceptible to proteinase kinase treatment than PrP^res^ found in scrapie-infected animals. Consequently, a larger fragment of the N-terminal sequence of BSE PrP^res^ (PrP 76–89 region) is deleted. The P4 antibody that recognizes the N-terminal end of the protein binds well only to PrP^res^ in scrapie and almost not at all to PrP^res^ in sheep with BSE. In contrast, monoclonal antibodies 6H4, 66.94b4, 505, 8G8, and R521, recognize the core region, and bind PrP^res^ in samples from both BSE- and scrapie-infected sheep; thus, WB with double antibody discrimination can easily distinguish BSE and scrapie [6,8,75,79]. Furthermore, the ratio of P4/core antibody is significantly higher for scrapie than for BSE [16,53,78,82]. Thuring et al. showed that the ratio of 66.94 b4/P4 monoclonal antibodies above 1.5 is a practical indicator of a serious suspicion of BSE in sheep since this ratio in samples from the sheep with BSE was much higher than 1.5 and differed significantly from the ratio seen in samples from scrapie-infected sheep (all remained below this value) [53]. This could be explanained by the fact that the epitope for mAb P4 is located near the N-terminal end of the PrP protein and is localized near the proteinase K cleavage site. It appears that an important part of the epitope for BSE recognition may be destroyed during the hybridization procedure, decreasing the ability of the antibody to bind to BSE PrP^res^. It could also be possible that differences in the protein folding during the techniques cause conformation differences, and this masks the epitope for BSE in sheep but has no effect on the epitope for scrapie PrP^res^ [78]. Thuring et al. and Stack et al. also revealed that the concentration of diglycosylated PrP^res^ was significantly higher in samples from BSE-infected sheep (over 70%) than in samples from sheep infected with scrapie (below 70%) [53,78]. Although glycoform profiling is a useful diagnostic tool does not always show a distinction between scrapie and BSE. Furthermore, this method of strain typing also shows considerable variation between laboratories. Therefore, it has been proposed that due to the high variability between blots, glycoform profiles should be obtained from repeated examination of samples across multiple blots using photo-imaging techniques to quantify band intensities. The inclusion of appropriate controls on each gel is also important in aiding the differentiation of the BSE in sheep samples [78,79].

TSE isolates can also be distinguished based on the proteinase K sensitivity of PrP^res^ since PrP^res^ associated with BSE in cattle or sheep is more sensitive to PK treatment than PrP^res^ found in scrapie-infected animals. Consequently, a large fragment of the N-terminal sequence of PrP^res^ is deleted by proteolytic treatment, resulting in the loss of the relevant epitopes (e.g., P4). The epitope recognized by the mAb P4 was specifically unexpressed in BSE-infected animals while remaining in scrapie-infected sheep [78]. Differential immunoblotting techniques based on the proteinase K sensitivity involving two specific antibodies directed against the carboxy- and amino- terminal ends of PrP have been developed [78,81,83].

Owen et al. developed a new strain-typing method that uses the thermostable protease thermolysin to distinguish BSE from natural scrapie isolates. Digestion resulted in different PrP^Sc^ cleavage patterns for ovine BSE and scrapie. WB analysis using the monoclonal antibody P4 revealed different PrP^res^ profiles for experimental BSE compared to those for natural scrapie. Scrapie samples produced bands with apparent molecular masses of approximately 36, 28, 23, and 19 kDa, while BSE samples formed poorly separated bands with apparent molecular masses of approximately 36 and 28 kDa. This test has several advantages. It does not rely on subtle differences in the molecular weights of PrP^res^ bands resulting from PK digestion of BSE and scrapie samples and does not require molecular weight and glycoform ratio determinations. Furthermore, it does not rely on the analysis of samples on multiple gels to allow the quantitative assessment of differences in the anti-PrP antibodies binding [84].

It was also shown that ovine BSE was more stable to guanidinium hydrochloride (GndHCl) than classical scrapie and that treatment with 3.5 mol/L GndHCl before digestion in combination with P4/core antibody binding ratios allowed discrimination of ovine BSE from scrapie [85]. More recently, the identification of additional C-terminal PrP^res^ product specifically recognized by a C-terminal antibody (SaF84) may contribute to the discrimination of scrapie and BSE [83]. There is evidence that there is a significant truncation of atypical scrapie PrP^res^ in the C-terminal domain compared to BSE and classical scrapie PrP^res^, and further, that the N-terminus is less truncated than BSE PrP^res^ [86,87]. 

Diagnosis and discrimination of BSE types via the Western blot method can be difficult when the signal is very strong or weak. A very strong signal does not permit accurate molecular mass measurements or distinguishing protein bands for glycoform profiling. A weak signal may show a single diglycosylated band or a weak signal for diglycosylated and monoglycosylated bands with the absence of an unglycosylated band [21,76]. In very weak positive samples, the ultracentrifugation step prior to loading the samples onto an SDS-PAGE gel considerably enhances the concentration of PrP^Sc^, leading to a much more intense Western blot signal [88]. The use of the correct brain sample is important for correct diagnosis. The preferred sample for immunoassay should be the medulla at the level of the obex. It was found that a direct Western blot of the cortex or cerebellum may yield negative results. If the sampled tissues are autolyzed, they can still be collected and tested. A positive result in such cases is still a valid result, but a negative test result cannot indicate a negative animal. Western blot has similar diagnostic sensitivity to IHC and remains the method, along with IHC, for confirming suspected cases of BSE. This method requires fresh or frozen material, which is not always available, and the ability to use these tests retrospectively may be limited, as formalin-fixed, paraffin-embedded tissues are most commonly used. Western blot also does not provide information on the neuroanatomical location of PrP^Sc^. However, Western blot, similar to IHC, can detect PrP^Sc^ in the brain of BSE-infected animals that do not yet show clinical signs or before vacuolation occurs. Accumulation of PrP^Sc^ can be detected at least 6 months before the onset of clinical signs [89]. 

## 7. EU-Approved Rapid Tests

There are tests of high sensitivity that can be performed within 24 h and are applicable to high throughput testing. These kits are based on Western blot, immunochromatography (lateral flow immunoassay), and enzyme-linked immunosorbent assay (ELISA). Several rapid tests have been developed for the screening detection of BSE in the central nervous system tissue of cattle. Tests officially approved for active surveillance activity within the EU are listed in Chapter C of Annex X to the TSE Regulation No 999/2001 and subsequent amendments. 

Currently, a European Union commission has approved six such tests:

- Prionics^®^-Check Western test,
- Prionics^®^-Check LIA test,
- Bio-Rad TeSeE SAP rapid test,
- Roboscreen Beta Prion BSE EIA Test Kit,
- IDEXX HerdChek BSE-Scrapie Antigen Test Kit, EIA,
- Prionics^®^-Check PrioSTRIP.

Only the following methods should be used as rapid tests for the monitoring of TSE in sheep and goats:

- Bio-Rad TeSeE SAP rapid test,
- Bio-Rad TeSeE Sheep/Goat rapid test,
- IDEXX HerdChek BSE-Scrapie Antigen Test Kit, EIA.

All these tests, apart from the IDEXX HerdChek tests, use unique features of PrP^Sc^, including its increased resistance to proteinase K digestion. Therefore, samples after homogenization are subjected to PK digestion, which digests the normal PrP^C^ but leaves protease-resistant prion protein PrP^Sc^. Anti-PrP antibodies are then used to detect the intact PrP^res^. The Prionics^®^-Check Western blot test was the first BSE rapid test used in large-scale epidemiological studies and was first approved by the Swiss authorities in 1998. In 1999, it was officially accepted by the EU as the only test with 100% sensitivity and specificity. It is based on an optimized Western blot procedure that enables the monitoring of the characteristic three-band pattern of the protein-resistant PrP^Sc^ fragment. Digested homogenates are subjected to gel electrophoresis, and then the Western blot is performed. The blot membranes are incubated with a monoclonal antibody with a high affinity for PrP to detect protease-resistant PrP^Sc^. This test uses mAb 6H4 as a primary antibody. The signal is visualized using the secondary antibody-alkaline phosphatase (AP) conjugate. The Prionics^®^-Check Western blot, like IHC, can identify animals with BSE that do not yet show clinical signs [50]. Wells et al. revealed that spongiform changes in experimental BSE were visible only at a very late incubation stage, shortly before the onset of clinical signs. Accumulation of PrP^Sc^ can, therefore, be detected at least 6 months before clinical signs appear [50,90]. The high affinity of the antibody and the unique properties of the buffers used in the Prionics^®^-Check test allow the test to be performed directly with tissue homogenates combining the reliability of the WB procedure with the speed needed for large-scale screening. A positive diagnosis of BSE is based on the detection of molecular weight and glycosylation patterns via specific antibodies; therefore Prionics^®^-Check is both a qualitative and quantitative test. 

**Figure 6:**
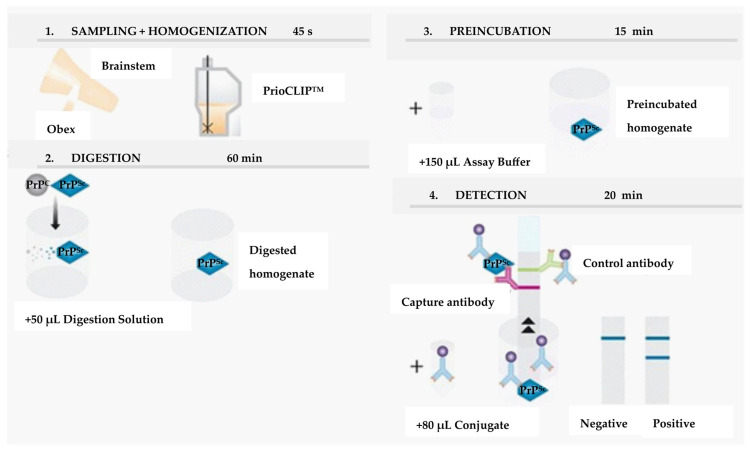
Principle of Prionics^®^-Check PrioSTRIP test. Adapted from Ref. [92].

The Prionics^®^-Check PrioSTRIP is a purely quantitative immunochromatographic assay that uses two different mAbs to detect PrP^res^. The Prionics^®^-Check PrioSTRIP follows a four-step protocol, consisting of homogenization, digestion with proteinase K, preincubation, and detection. If PrP^Sc^ is present in the homogenates, it binds to the conjugate, which is a monoclonal antibody labeled with latex beads. By imersing the PrioSTRIP^®^ into the sample-mixture, the flow through the membrane is initiated. Prion-conjugate complexes are retained at the test line by the second (capture) antibody. The uncomplexed conjugate is bound at the control line that serves as the assay control. The test is purely quantitative. A negative result is defined by the observation of the control line (C) and the absence of a line in the test region (T). An initial reactive result is characterized by two lines both the test and the control line are seen (Figure 6). 

The test is novalid if no lines are visible or only the test line is visible. Interpretation of results is either visual or using the computerized scanning software (PrioSCAN ^®^ software), which converts the blue lines on the strips into digital data. The results obtained with the Prio-SCAN^®^ are reported as Relative Density Units (RDU). The sample is negative when the test line value is lower than the cut-off and the control line is present, while the sample is positive when the test line value is above the cut-off and the control line is visible. Manual interpretation of the combs would not be useful at low positive tissue concentrations. Visual perception and personal biases between different readers are likely to be more subjective than in an electronic-based system, where biases are eliminated [8,91,92]. All samples found to be an initial reactive must be retested in duplicate, and the results should be analyzed by two readers.

Prionics^®^-Check LIA test, Bio-Rad TeSeE SAP rapid test, Roboscreen Beta Prion BSE EIA Test Kit, and IDEXX HerdCheck BSE-Scrapie Antigen Test Kit, EIA represents ELISA methods that provide qualitative results relative to a cut-off value. Generally, in this detection system, the PrP^Sc^-specific ligand (captured monoclonal anti-PrP antibody or non-biological ligands) is immobilized in the wells of the plate, and test samples after digestion with proteinase K are incubated in the wells. If PrP^Sc^ is present, it binds to the ligand at the bottom of the plate wells. Then, after washing the wells, a detection antibody labeled with an activating enzyme is added. This second antibody binds to PrP^Sc^ and stains the substrate when PrP^Sc^ is present. The result is read as an optical density (OD) value. Samples with an optical density lower than the cut-off value are considered negative, while samples with an optical density higher than or equal to the cutoff value are considered positive. ELISA enables simultaneous analysis of more samples than Western blot, which is its main advantage. 

The Roboscreen BetaPrion^®^ BSE EIA Test and Bio-Rad TeSe SAP Kits consist of two modules, the Purification Kit, which includes the purification tools, and the Detection Kit, which is based on a sensitive ELISA. Both tests use two different mAbs against two different epitopes of PrP^Sc^. However, the Prionics^®^-Check LIA is a luminescence immunoassay, which uses two monoclonal sandwich antibodies, one to capture the proteinase K-resistant fragment and the other to detect it. The detection antibody is bound to horseradish peroxidase, which emits light upon exposure to a chemiluminescent substrate. The amount of light emitted correlates with the number of PrP in the sample. In the Prionics^®^-Check LIA test, the values obtained by the plate luminometer are given as Relative Light Units (RLU) and calculated by the Prionics^®^-Check LIA Analysis Software to identify positive and negative results [93,94]. 

In contrast to the previously described test, the IDEXX^®^ HerdCheck test does not need digestion with proteinase K since the test relies on conformational detection technology using specific aggregate-captured ligand on a dextran polymer (Microsens Biotechnologies, London, UK) [8,95]. Furthermore, it uses only one mAb. Once the sample is applied to the plate, the disease-associated conformer binds to the immobilized ligand with high affinity. After washing and incubation with conditioning buffer, the captured antigen is detected using a prion-specific antibody that has been conjugated to horseradish peroxidase (HRPO). After washing, peroxidase substrate is added. The color development is related to the relative amounts of PrP^Sc^ captured by the ligand immobilized in the well of the microplate. The interpretation of sample results is based on the sample absorbance. IDEXX offers three approved variants of BSE methodology for the HerdCheck test kit, the standard, short, and ultrashort tests. Those three protocols have equivalent performance but differ in the incubation conditions (duration of incubation, temperature, and agitation conditions) for critical steps of the protocol, i.e., initial incubation, capture plate incubation, and conjugate incubation [95].

In addition, the IDEXX HerdChek and Bio-Rad TeSe Kits are developed for the detection of PrP^Sc^ in post-mortem brain (obex preferred) tissues from bovines and small ruminants affected by BSE, scrapie, and chronic wasting disease (CWD). The difference in the performance of these tests toward scrapie and BSE infection may depend on the PK sensitivity, the choice of the primary anti-PrP antibodies, or both. It is known that PrP^res^ associated with BSE is more sensitive to PK treatment than PrP^res^ found in scrapie-infected animals. As a consequence, a large fragment of the N-terminal sequence of PrP^res^ is deleted by proteolytic treatment, leading to a loss of the relevant epitopes (e.g., P4). Thus, the epitope in the N terminus of the PrP^res^ recognized by the capture antibody is specifically unexposed in BSE-infected animals while remaining in scrapie-infected sheep [78]. Thus, in such ELISA tests, the capture antibody, immobilized in the solid phase, recognized an epitope in the N terminus of the PrP^res^, whereas the tracer antibody binds to the C-terminal moiety [80,96]. Although these EU-approved tests were developed for the detection of BSE and scrapie, a study performed by Meloni et al. revealed that the IDEXX test was significantly more sensitive (97%) than the Bio-Rad tests, which showed sensitivity > 80%. The IDEXX test was able to detect 100% of samples originating from BSE-infected goats and goats with clinical scrapie but missed 8% of samples from preclinical scrapie-infected goats. Bio-Rad TeSe tests also were able to detect all samples from goats with clinical scrapie but missed 15% to 25% of samples from clinical BSE-infected goats and up to 33% of samples from preclinical goats infected with scrapie. This suggests that BSE infection in goats may be underestimated in regions where Bio-Rad TeSe tests are used [97].

Size (the amount of brain material) and anatomical location of tissue samples on which the test must be applied must be strictly controlled to obtain optimal results. Therefore, the sampling and processing of brain tissue for use in rapid tests should be carried out according to the manufacturer’s instructions. However, Hagiwara et al. revealed that the usage of specimens of the brain region not specified by the protocols could also be used when rapid tests were used [7]. Furthermore, Smith et al. revealed that retina samples tested by the EIA HerdChek test were useful for the identification of TSE-infected animals with clinical signs of disease but not in TSE-infected animals that did not have clinical signs of the disease [98]. While correct sampling is important, tissue quality seems to be less important. If sampled tissues are autolyzed, they can still be taken and tested, which is important in any surveillance or diagnostic program [99,100]. In field conditions, brainstem samples are often autolyzed and contaminated with bacteria and other debris (feces, soil, hair). Obviously, these factors have an impact on the quality of the sample and affect the performance of the diagnostic test and the results generated [88]. Meloni et al. revealed that four rapid tests (Enfer TSE Kit, Bio-Rad TeSeE test, Prionics^®^-Check-LIA test, and IDEXX HerdCheck BSE Antigen Test Kit EIA) can be considered as well-running diagnostics tools regardless of tissue quality [100]. Wear et al. revealed that three rapid tests (Prionics^®^-Check Western blot, Platelia, and Enfer) also were able to detect PrP^Sc^ in autolyzed tissues. When autolysis was caused by storing samples under controlled conditions, the Prionics^®^-Check Western blot was able to detect positive scrapie and BSE samples after storage for 7 days at 37 °C [59,99].

Rapid tests are quick, reliable, and allow the diagnosis of a high number of samples; therefore, these tests are now the principal screening tools for active surveillance. Such tests provide a preliminary diagnosis and according to Annex X of the TSE Regulation, all the samples for which the result of the rapid test is positive or inconclusive should immediately be subjected to confirmatory examination by IHC or Western blot, demonstration of characteristic fibrils by electron microscopy, or by the combination of different rapid tests. A combination of two rapid tests is allowed for primary screening and subsequent confirmation. Rapid tests are highly reliable but sometimes produce initially false positive results. As was shown, several bacteria isolated from the autolyzed material, which were initially reactive, exhibit the characteristics of biofilm-forming bacteria, and this extracellular matrix might play a role in preventing the complete digestion of PrP^C^ in these samples and resulting in a positive signal on rapid tests [88,101]. 

Furthermore, it was noted that the *E. coli* chaperonin GroEL induces conformational changes and aggregation when mixed with recombinant PrP, leading to an increase in beta-sheet content and moderate protease resistance [102]. It has also been shown that lipopolysaccharide (LPS) of Gram-negative bacteria causes an increase in beta-sheet content in recombinant PrP, which is more similar to the structure of PrP^Sc^ [103]. These results might call into question the proposal established by the OIE, which dictates that the same two results from two different rapid tests are sufficient for official confirmation of BSE. Therefore, according to the guidelines from the European Union Reference Laboratory (EURL), one of the two rapid tests must be a Western blot. All tests must be scientifically proven and rigorously validated by the European Union. According to EFSA requirements, new tests will only be approved for evaluation if the LOD of these new tests is greater than 2 log_10_ compared to the most sensitive test [93].

In case of a confirmed positive case of BSE in cattle, further differential tests are obligatory to determine whether the BSE is classical or atypical (H-type BSE or L-type BSE). The exact rules for the diagnostic tests and diagnostic methods to be used are laid down in Annex X of the TSE Regulation. All currently recognized forms of BSE are detectable by EU-approved rapid tests. Hagiwara et al. revealed that rapid ELISA tests (TeSeE^®^, FRELISA^®^, and NippiBL^®^) were suitable for detecting C-, L-, and H-BSE prions [7]. Gray et al. tested three rapid tests (a PrPSc-conformation-specific ELISA (confELISA), a standard sandwich enzyme-linked immunosorbent assay (stndELISA), and an immunochromatographic assay), for atypical BSE PrP^Sc^ and showed that all assays were able to meet the same requirements that the European Food Safety Authority set when evaluating the tests for PrP^Sc^ type C BSE [104]. Meloni et al. revealed that the IDEXX HerdChek^®^BSE-scrapie test with a short protocol test showed the highest sensitivity for all types of BSE (C-, L-, and H-type). The Prionics^®^-Check WESTERN, the IDEXX^®^ HerdChek BSE-scrapie test with ultra-short protocol, and the AJ Roboscreen^®^ BetaPrion tests had similar sensitivity, while the Bio-Rad^®^ TeSeE™ SAP, the Roche^®^ PrionScreen and the Prionics^®^-Check PrioSTRIP showed the lowest sensitivities for all the BSE types. Bio-Rad^®^ TeSeE™ SAP and the Prionics^®^-Check PrioSTRIP displayed the lowest analytical sensitivity, notably for L-BSE. Despite these differences, the detection limit of all these tests was set within a 2 log_10_ range of the best test, thus meeting the European Food Safety Authority requirement for BSE surveillance programs [8].

## 8. The Immunofluorescence Assay (IFA), Flow Cytometry, and Immunofluorometric Assay (IFMA)

Since PrP^Sc^ and PrP^C^ share the same primary structure, most of the antibodies used against the PrP molecule are unable to distinguish these two proteins. Taraboulos et al. reported that pretreatment of fixed cells with guanidinium salts considerably increases the PrP^Sc^ signal after immunofluorescence staining [105]. Although the mechanism of specific PrP^Sc^ detection by this pretreatment remains unclear, this method has been used to detect PrP^Sc^ in cells or frozen tissue sections by immunofluorescence assay (IFA) and flow cytometry [106,107,108,109]. Specific detection of PrP^Sc^ can be performed using pan anti-PrP monoclonal antibody 132, which recognizes the region adjacent to the most amyloidogenic region of PrP, AGAAAAGA. Since most pan-PrP antibodies cannot distinguish PrP^Sc^ from PrP^C^, manipulations such as detector gain or exposure time are needed to set a threshold level at which PrP^C^ signals are below the detection limit [108,109]. In fact, pretreatment with GdnHCl does not prevent the detection of PrP^C^ in uninfected cells [105,108]. Alternatively, PK treatment is required prior to pretreatment with GdnHCl to completely abolish PrP^C^ signals [105,108]. However, PK treatment affects cell architecture, making analysis difficult. 

In the IFA, the cells on the plate or cut sections are fixed by 4% paraformaldehyde (PFA) so that the proteins in the cells are poised for antibody binding. After removal of the fixation solution, the remaining paraformaldehyde is neutralized with 0.1 M glycine, and then the cells are permeabilized by a proper detergent such as Triton X-100. For the detection of PrP^Sc^, cells are pretreated with 3–5 M guanidine thiocyanate (GdnSCN) to denature PrP^Sc^. After blocking, the cells are incubated with primary antibodies in the same blocking solution. Then, after washing, the cells are incubated with Alexa-Fluor-conjugated secondary antibodies in the dark. The samples are then stained and mounted, and examined using a confocal fluorescence microscope or a laser scanning microscope [110].

For flow cytometry of cells prepared from the brain, it is necessary to use density-gradient centrifugation to remove cell debris from samples, as excess debris prolongs the measurement time by a cell sorter. PrP^Sc^ is hydrophobic and, therefore, more likely to bind to biomolecules nonspecifically, which can cause a false positive result in the flow cytometric analysis. Antibodies other than 132 could not be used to distinguish prion-infected cells from uninfected cells with flow cytometry. The difference between mAb 132 and other mAbs (106, 31C6, and 44B1) is the lack of PrP^C^ signals from uninfected cells; thus, the absence of PrP^C^ signals rather than the intensity of PrP^Sc^ signals is critical for the clear discrimination between negative and positive cells [88]. Recently, cell-based ELISA has been developed in which PrP^Sc^ can be directly detected from permanently infected cells using anti-PrP mAb 132. This new cell-based ELISA can discriminate prion-infected cells from prion-uninfected cells without the need for cell lysate preparation and PK treatment. Such ELISA is a practical and simple method for the primary screening of anti-prion compounds [111,112]. 

The principles of flow cytometry are now widely used for automated endpoint analysis in microwell immunofluorometric assay (IFMA), which allows simultaneous detection of multiple analytes (multiplex). IFMA uses multiple sets of microbeads sets, each with a distinct internal fluorescent signature (fluorescent microspheres) and each paired with one of a range of capture antibodies with different epitope specificities. A mixture of these bead/antibody combinations is easily differentiated by automated flow cytometry [113,114]. Tang and co-workers developed simple multiplex immunoassays to detect TSEs and differentiate scrapie and BSE [115]. In this test, samples after PK digestion and denaturation were incubated with two capture antibody beads, 12B2 and 9A2, specific for different N-terminal regions of PrP^res^ in combination with the reporter antibody Sha31. The 12B2 epitope is foundonly in PrP^res^ derived from classical scrapie. Therefore, PrP^res^ from both BSE and scrapie samples produced significant binding to 9A2 and low binding of BSE samples to 12B2. Therefore, a low 9A2/12B2 binding ratio indicates scrapie, while a higher ratio indicates BSE. Ratios for scrapie ranged from 0.4 to 1.8, while for ovine and bovine BSE, it ranged from 2.9 to 19. This method showed high predictive value (100%) when applied to brain samples from control animals, sheep naturally infected with scrapie or experimentally infected with BSE, and cattle infected with BSE [115]. 

Tang et al. extended the IFMA assay to differentiate atypical scrapie, classical scrapie, CH1641 scrapie (atypical scrapie), and ovine and bovine BSE, incorporating a third core-specific antibody 94B4. The 12B2 and 94B4 antibodies bind PrP^res^ of classical scrapie. However, because atypical scrapie PrP^res^ has a truncated C-terminal end that does not bind 94B4, only the 12B2 binding region is reported by IFMA. The degree of N-terminal truncation of BSE PrP^res^ dictates that only 94B4 binding is reported since the 12B2 epitope is significantly cleaved by PK digestion. This test provides an important advance in diagnostic capability because it allows the differentiation of BSE from the unusual CH1641-type scrapie. Using Western blot, these TSE isolates remained indistinguishable because the PrP^res^ CH1641-type scrapie shares some molecular features with BSE PrP^res^ [116]. This novel triplex IFMA enables simultaneous detection and discrimination of four TSE types. The method is sensitive and can be applied in large-scale testing, so it is suitable for both screening and confirmatory differential diagnosis. In addition, IFMA is less labor-intensive than most other methods. However, despite these advantages, IFMA is a rarely used method for detecting BSE infection.

## 9. Scrapie-Associated Fibrils (SAFs)

Scrapie-associated fibrils (SAFs) have been recognized as specific ultrastructural markers for TSEs, found in extracts of the brains of infected animals. They were not found in the brains of normal healthy animals. SAFs were first characterized in brain extracts from affected mice and hamsters experimentally infected with scrapie [117]. These fibrils consist of protease-resistant PrP^Sc^ and can be extracted from fresh or formalin-fixed tissue as well as from frozen and autolyzed tissue. Biochemical SAF extraction techniques are generally based on extracting a crude mitochondrial pellet, which is sub-fractioned to obtain a fraction of synaptosomal plasma membranes. The technique is based on extraction with the N-lauroylsarcosine detergent, followed by differential centrifugation and proteinase K digestion. The final pellet of the brain extract is dispersed in sterile deionized water and placed on dried formvar/carbon-coated grids. After blocking, the samples are incubated with a primary antibody and, after washing, exposed to colloidal gold-conjugated immunoglobulin. The grid is then negatively stained with 2% phosphotungstic acid (pH 6.6). SAFs are detected by negative-contrast transmission electron microscopy (TEM). Electron microscopy observation should reveal fibrils with a simple or double helix structure of 100–500 nm in length. A five-point scale was used for SAF scoring. If no fibrils were seen after a 20 min time observation, the sample was assumed to be negative. If only one to two fibrils were seen during the observation period, the score was 1. The scores of 2, 3, and 4 were reported when approximately 5, 25, and 100 fibrils were observed, respectively [118,119,120,121]. SAF visualization by electron microscopy has been confirmed as a highly sensitive technique; however, the Western blot method is more sensitive [122]. Currently, SAF is rarely used, but it may be a method of choice when only severely autolyzed or formalin-fixed samples are available [62]. The fibrils seems to be extensively distributed across the brain, but studies have shown that if only one region of the brain is tested, there may be a problem with false negative results [122]. Furthermore, a gradual decrease in fibril yield was observed when the final extracted pellets were stored at 4 °C [123]. Positive results by the SAF test alone have been treated with caution since the appearance of SAFs depends heavily on the purification scheme. Therefore, the positive results may perhaps be due to the concentration of foci of PrP^Sc^ by the SAF extraction process [124]. 

## 10. In Vivo Bioassay

A very specific and sensitive method of diagnosing BSE is experimental infection of laboratory animals. Samples indicative of BSE or inconclusive for BSE, after secondary molecular testing, should be further analyzed by in vivo bioassay for final confirmation. In these methods, animals are injected with very small amounts of PrP^Sc^ and the incubation period and clinical signs indicating the presence of infectious material are monitored. After the death of the animal, disease development is confirmed using classic techniques such as histology, IHC, and Western blot. Lesion profiles, patterns of pathological lesions, and biochemical features of the PK-resistant PrP^Sc^ are evaluated; thus, a bioassay is considered the gold standard for distinguishing classical ovine scrapie and ovine BSE. 

Initially, in vivo bioassay was done in wild-type rodent models (mice, hamsters, bank voles), resulting in extremely long incubation times. The conventional RIII mouse has been widely used to detect bovine prions in classical BSE pathogenesis studies in cattle but showed relatively reduced sensitivity to low-titer inoculum since bovine prions have to cross the species barrier between mouse and cattle [125]. Using this assay, BSE was detected only in the central nervous system, retina, spinal cord, and distal ileum of experimentally infected calves. No BSE was detected in other tissues [126]. To circumvent this problem, bovine prion protein (PrP) transgenic mice (Tgbov XV mice) have been successfully generated to provide facilitated transmission of BSE prions. Transgenic mice expressing bovine PrP^C^ on a PrP-deficient background have been shown to exhibit symptoms between 230 and 340 days after BSE infection, depending on the inoculum and the infected transgenic mouse line [108]. Tgbov XV mice were found to be more than 10^4^ times more sensitive than RIII mice and ~10 times more sensitive than cattle in detecting classical BSE. Therefore, Tgbov XV mice are the most valuable tool for testing the BSE infectivity of bovine tissues and bodily fluids [126]. Transgenic mice for bovine PrP^C^ have been used to demonstrate the specific characteristics of all BSE types, demonstrating that all these BSE types can be distinguished in bovinized mice [127]. In addition, gene-targeted Tg mice expressing bovine PrP, which are produced by gene replacement, do not suffer from any unfavorable phenotypes that are associated with overexpression or ectopic expression of the transgene in standard transgenic lines, and can, therefore, better reflect what happens in nature [127]. Advances in transgenic mice contributed to the development of more sensitive and faster bioassay models. However, in vivo bioassay is too labor-intensive and time-consuming to be used in routine high-throughput screening. In addition, the results are not always easy to interpret. Prion replication during the incubation phase proceeds very slowly and it can take several months or even years before a detectable amount of PrP^Sc^ accumulates in the brain. Furthermore, a bioassay is insensitive, especially with low-dose inoculum. Several problems also arose when autolytic samples were used for inoculation. Such samples should be heat-treated to inactivate microbial contamination and should be diluted to reduce toxicity. Despite such procedures, a high number of mice may die, as demonstrated by Sarasa et al. [62]. 

Recently, Thackray et al. revealed that transgenic Drosophila for bovine PrP were highly susceptible to BSE prion infectivity and established Drosophila as a new host to mammalian bioassay prions [128]. Bovine PrP in Drosophila allowed the detection of classical BSE, atypical L- and H-type BSE, and sheep-passaged BSE at levels 10^6^-fold lower than that obtained in bovine PrP mice bioassay. The bioassay with bovine prions on the fly could be carried out within 7 weeks, in contrast to the bioassay with prion on mice, which required at least 1 year to evaluate the same inoculum. In addition, bovine PrP Drosophila could detect classical BSE at a level 10^5^ times lower than PMCA. These data indicate that PrP transgenic Drosophila represents a novel, easy-to-perform prion bioassay for the efficient and sensitive detection of mammalian prions, including those with known zoonotic potential [128]. 

It should be noted that, especially in rodents, the phenotypes are generally not specific to the host, but rather mouse-line-specific. This means that phenotypes between different mouse lines should not be directly comparable even within wild-type mice due to the polymorphisms in the murine PrP and other genetic factors [129]. Bioassay also provides information about the infectivity of PrP^Sc^, as end-point titration allows estimation of the degree of prion infectivity in different materials and tissues. Bioassay has the highest sensitivity when conducted in homologous species. 

## 11. Protein Misfolding Cyclic Amplification (PMCA)

**Figure 7:**
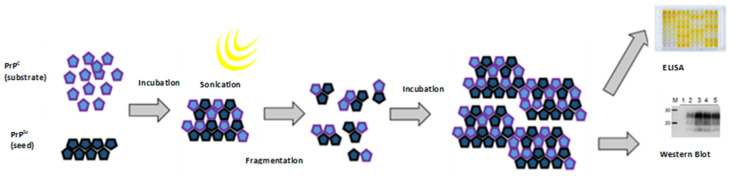
Principle of protein misfolding cyclic amplification (PMCA). Adapted from Ref. [79].

The use of a PK-based test for routine testing raises the fear of the reappearance of BSE, as 80% of the tested PrP^Sc^ is believed to be PK-sensitive. This can lead to the generation of false negative results. In addition, PK-based methods have been developed for the analysis of brain tissues, so they can be only used for postmortem diagnosis. Since PrP^Sc^ accumulates in extremely low amounts in other tissues and biological fluids, therefore, one important objective in prion research was to develop a highly sensitive alternative method to detect minute amounts of PrP^Sc^. In 2001, Soto et al. described a new type of in vitro technique called protein misfolding cyclic amplification (PMCA), in which it is possible to stimulate prion replication in the tube in an accelerated mode [130]. PMCA is a cyclic process, and each cycle is composed of two phases: incubation and sonication. During the first phase, the tested sample is mixed with the substrate as the source of PrP^C^ and incubated at 37 °C to induce the formation of PrP^Sc^ polymers/fibrils. Minute amounts of PrP^Sc^ (termed the seed) in samples derived from infected animals serve as a template for the PrP^C^, which adapts to the conformation of PrP^Sc^ and incorporates it into the growing ends of PrP^Sc^. In the second phase, the sample is sonicated to break the elongated polymers into shorter fragments providing new templates (seeds) for subsequent conversion. Therefore, with each cycle, there is an exponential increase in the number of seeds. The cyclic nature of the system enables as many cycles as required to reach the amplification state needed to detect PrP^Sc^ in a given sample. After amplification, samples are digested with proteinase K and PrP^res^ is detected by immunological methods such as Western blotting or ELISA (Figure 7) [131]. 

Originally, normal, healthy brain homogenate originating from the same species as the infectious sample to be amplified was used as the best substrate for high-efficiency amplification. Furthermore, detergent-resistant membrane (DRM) or lipid-raft fraction of the plasma membrane was considered a good alternative substrate to the whole brain homogenate [131,132]. More recently, PrP^C^ was purified from brain tissue or cultured mammalian cells, and the recombinant PrP (rPrP) expressed in bacterial cells replaced brain material, enabling faster and simpler detection than conventional PMCA methods. The substrate concentration could therefore be determined more precisely and efficiently in rPrP-PMCA than in classical PMCA. Transiently or stably transfected knock-out-PrP (PrP-KO) cells overexpressing different PrP^C^ transgenes could provide useful substrates for PMCA [133,134,135,136,137]. In order to create a versatile and convenient PMCA procedure, unconstrained by the availability of substrate sources, Mays et al. developed PMCA using cell lysate that express cellular PrP^C^. PrP^Sc^ was efficiently amplified with the lysate of many cell lines of neuronal or non-neuronal origin, demonstrating that cell lysate in which PrP^C^ is abundant serves as an ideal substrate source for PMCA. Their results suggested that cell lysate can replace animal organ-derived material for PMCA amplification. They also noted that the PrPC abundance is a critical factor in guaranteeing reliable PrP^Sc^ amplification [138]. More recently, the addition of a synthetic polyanion, polyadenynic acid (polyA), and RNAs was found to increase PrP^Sc^ amplification in the PMCA, but spontaneous PrP^Sc^ production was observed after several rounds of reaction, which makes the detection of genuine PrP^Sc^ in specimens difficult [139,140]. The efficiency of BSE PrP^Sc^ amplification was significantly increased when amplification was carried out in the presence of sulfated dextran (DSP) compounds at 37 °C. The DSP-PMCA method was 10^5^ times more sensitive than the bioassay, and this method was capable of amplifying marginal amounts of PrP^Sc^ from peripheral tissues of BSE-infected cattle [141]. Furthermore, ceramic and Teflon beads greatly improved the efficiency of PMCA. Beads are thought to promote efficient fragmentation of PrP^Sc^ polymers, thereby enhancing the number of seeds available for conversion [142,143]. Using the so-called miniaturized bead-PMCA (mb-PMCA) protocol, a single 48 h round was sufficient to achieve amplification to a level detectable by the conventional Western blot method. The sensitivity of mb-PMCA was 10^4^–10^5^ times higher than bioassay [142]. Supplementing PMCA with electrical energy (ePMCA) has lead to significant improvements in the efficiency and reproduction rate of prion conversion and in the sensitivity of PrP^Sc^ detection. It remains unclear how electricity affects prion conversion, but it is hypothesized that the incubation process with electricity may help accelerate the rate of efficient PrP^C^ to PrP^Sc^ conversion, making prion conversion more optimal [144]. For samples containing very small amounts of PrP^Sc^, serial PMCA (sPMCA), in which the amplified sample is diluted into the fresh substrate at each round, is highly recommended [131]. sPMCA has very high sensitivity (97.2%) and specificity (100%) for BSE and can be used to distinguish BSE prion protein from the scrapie prion protein. Ovine BSE was amplified using substrates from sheep with VRQ/VRQ or AHQ/AHQ genotype, while classical and atypical scrapie did not amplify in any of these substrates. Therefore, the use of a combination of these substrates encourages the amplification of BSE over scrapie, even when scrapie-positive brain materials were present at a 100-fold excess over BSE-infected materials [145]. The sPMCA assay was found to be better than Western blot and ELISA tests in detecting BSE in sheep in the presence of a large excess of scrapie-infected material. Therefore, sPMCA could be used to monitor the status of animals with mixed infections where the levels of BSE are low [146]. Chen et al. described a means of achieving quantitative estimates of prion seeding activity using PMCA reactions, termed quantitative PMCA (qPMCA) [147]. In this assay, a single dilution of the sample is tested in a serial PMCA reaction, and the relative seeding activity is calculated from the number of serial PMCA rounds needed to detect a positive response. 

The composition of the conversion buffer is very important, as it has been found that even small changes can drastically affect the efficiency of the amplification process. Divalent metal ions, such as copper and zinc, inhibit PrP^Sc^ conversion [148]. For best results, a pH between 7.0 and 7.3 is necessary. The proper preparation of the substrate and starting material as a source of PrP^C^ is critical. The use of previously denatured samples using chaotropic agents or ionic detergents at high concentrations should be avoided. The use of formalin-fixed samples is also not recommended [131]. Furthermore, either the samples to be amplified or the substrate may contain inhibitory PMCA (plasminogen, cations). Since it is extremely important to eliminate such molecules, samples such as blood, and tissue samples containing high amounts of blood, saliva, milk, urine, and feces require pretreatment before the amplification process. Sonication conditions are also very important. Appropriate regulation of sonication dramatically accelerates PrP^Sc^ amplification allowing the detection of <1 LD_50_ (50% lethal dose) of PrP^Sc^ in the diluted brain homogenates after only one or two rounds of reaction [149]. 

The development of in vitro PMCA assay opened a new era in prion research. PMCA provide the potential to enhance existing methods by increasing the amount of PrP^Sc^ in the sample. Combining an in vitro prion conversion strategy with any of the highlysensitive detection methods, the early detection of BSE may be achieved. PMCA is a promising method for prion diagnosis in biological tissues or fluid samples where the concentration of PrP^Sc^ is well below the detection limit of currently available analytical methods. PMCA was able of amplifying extremely low amounts of PrP^Sc^ from the saliva, lymph nodes, palatine tonsils, muscular tissues, and ileocecal regions of BSE-infected cattle [141] and in urine, cerebrospinal fluid (CSF), and plasma from macaques experimentally infected with classical BSE and atypical L-BSE [150,151]. The presence of PrP^Sc^ in the blood of BSE-affected cattle has not been confirmed, indicating that BSE is not transmitted through the blood [141]. 

It was shown that the PMCA-generated form of PrP is fully infectious and shares similar biochemical, structural, and biological properties with the original seeds that serve for amplification [142,152]. This provides evidence that the in vitro conversion during PMCA is similar to the events that occur in vivo, leading to disease and death. This phenomenon provides an opportunity to examine many aspects of prion biology (develop prion decontamination procedures, identification of inhibitors and drugs to prevent PrP^Sc^ formation and elimination, quantification of the extent of prion contamination in medical or environmental materials, and interspecies infectivity). PMCA was used to assess the possibility of atypical BSE crossing the species barrier into humans. Neither H- nor L-type BSE induced significant conversion in PMCA in contrast to C-type BSE, indicating that atypical forms of BSE pose a poor zoonotic threat [153,154]. These findings contradict previous results that showed that L-type BSE was able to produce infectivity in a humanized transgenic mouse model overexpressing the human prion protein [155]. 

The PMCA has been automated by utilizing a programmable microplate horn sonicating system leading to high throughput and highly efficient amplification of PrP^Sc^ [131,142]. In just a few weeks, this technique can replicate PrP^Sc^ and converse prion strain properties in a way that replicates the time-consuming in vivo studies that can take several years. Furthermore, automatic PMCA overcomes one of the main disadvantages of manual PMCA, i.e., cross-contamination. However, despite the advances made with PMCA, the technique has some drawbacks. The need for sophisticated sonication equipment leads to different results in different laboratories. PMCA requires serial rounds of amplification to reach maximal sensitivity, which can be time-consuming. Furthermore, a higher number of rounds raises the possibility of generating false-positive reactions [156]. Preparation of substrates by expression/purification of native PrP^C^ from animal tissues and cell lines, as well as recombinant PrP from bacterial cells, requires additional, laborious, and time-consuming purification steps. Another limitation is the need for a separate readout system, such as WB, which extends the total time needed for diagnosis.

Chang et al. described a method combining PMCA-like in vitro PrP^Sc^ amplification with aggregation-specific (AS) ELISA and a fluorescence-catalyzed T7 RNA polymerase (FACTT) amplification technique, named Am-A-FACTT [157]. In this method, plasma is mixed with healthy brain homogenate and undergoes amplification. Newly formed PrP^Sc^ aggregates are then captured by an aggregate-specific mAb in AS-ELISA in combination with FACTT, where detection is via a biotin-conjugated DNA template. After transcription of the DNA template into RNA, an RNA-intercalating dye is added, and the intensity of the emitted light is measured by a fluorometer. Am-A-FACTT was used to detect PrP^Sc^ in asymptomatic mule deer infected with chronic wasting disease (CWD) and in the blood of scrapie-infected mice but has not been used to study PrP^Sc^ from BSE [157]. 

## 12. Real-Time Quaking-Induced Conversion (RT-QuIC)

Over the last decade, PMCA has been progressively replaced by the quaking-induced conversion (QuIC) assay. QuIC, developed by Atarashi et al. differs from PMCA in that sonication has been replaced with automated shaking for the conversion of recombinant PrP^C^ substrate [158]. During the incubation phase, PrP^Sc^ acts as a seed, inducing a conformational change of substrates into amyloid fibrils and the formation of PrP^Sc^ polymers. Intermittent vigorous shaking promotes the fragmentation of generated polymers, forming more reactive seeds, and speeding up the conversion process. In the QuIC, soluble bacterially expressed recombinant PrP (rPrP) is used as a substrate (PrP^C^ source). Thus, the QuIC method solves the problem of using the brain as a substrate for amplification. The most suitable rPrP substrate may vary depending on the bodily fluids or tissues under study. Many substrates have been used for QuIC reactions, including hamster-sheep chimera [159], hamster (23–231 and 90–231) [160], mouse (23–231) [161], human (23–231) [162], sheep (25–234) [163], deer (24–234) [160], and elk (24–234) [164,165]. The bank vole has an extremely weak species-specific barrier to prion transmission, as it is susceptible to multiple PrP^Sc^ strains from multiple species, including sheep, humans, hamsters, and deer. Therefore, bank vole rPrP is used as a universal substrate for QuIC [166,167,168]. It was also shown that lyophilized bank vole rPrP could be utilized for RT-QuIC, since the lyophilization and resolubilization step causes minimal protein loss, with no noticeable change in secondary structure or unfolding temperature [169]. 

**Figure 8:**
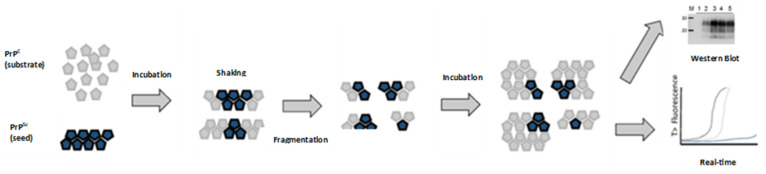
Principle of real-time quaking-induced conversion (RT-QuIC). Adapted from Ref. [79].

The original standard QuIC (S-QuIC) format required a time-consuming Western blot. One of the major advantages of QuIC is the replacement of Western blotting with a real-time fluorescent color reaction based on a fluorescent amyloid-sensitive thioflavin T dye (ThT), which minimizes the time required to detect protease-resistant rPrP fibrils (rPrP^res^) (Figure 8). ThT undergoes a fluorescence shift upon binding to amyloid fibrils from excitation/emission maxima of 342/430 to 442/482 nm. The ThT dye does not fluoresce significantly at excitation/emission maxima of 442/482 nm in the absence of amyloid fibrils. Therefore, the background in the assay is extremely low. The incorporation of ThT into elongating fibrils is monitored on a multi-well plate in real-time by reading ThT fluorescence over time. 

The reactions are carried out in multi-well plates that are interrupted by shaking in a fluorescence plate reader, providing a relatively high-throughput and viable system for prion disease diagnosis. RT-QuIC is similar to the amyloid seeding assay (ASA). It differs only in the use of an ionic detergent, such as sodium dodecyl sulfate (SDS) instead of a denaturant. Atarashi et al. indicated that fibrils generated in the presence of SDS were significantly thicker and larger than those generated in its absence. Therefore, a low concentration of SDS is preferred for the optimal prion detection and to reduce the rate of false-positive responses [134]. The use of a guanidine-HCl-free buffer is recommended as it reduces the risk of false positive reactions and enhances the sensitivity of the method. The presence of salt is critical for cell-free conversion. It was shown that the sensitivity of RT-QuIC was maximal at 500 mM NaCl at pH 7.4 [169]. Lowering the pH elongated the lag phase of the method, so neutral pH is optimal [148,170,171]. A higher incubation temperature (55 °C) or more vigorous shaking conditions (1100 rpm) can also increase the sensitivity of the reactions, but continuous shaking without a rest period is not recommended because it induces spontaneous reactions resulting in false-positive signals [170].

Quantitation of relative levels of prion seeding activity can be achieved using endpoint diluted samples, analogous to endpoint dilution titrations in animal bioassay. Sample dilutions are used as seeds, and the seeding dose (SD_50_) is defined as the minimum seeded amount that causes 50% of RT-QuIC reactions to be positive [160]. To obtain SD_50_ values for a single prion strain in RT-QuIC, a serially diluted standard sample (serially diluted prion-infected reference brain homogenate) and serially diluted undetermined samples are required. Thus, this method requires multiple repeats to obtain the percentage of positive reactions for each dilution. Based upon the quantitative correlation between prion seed concentration (prion titer in sample) and the lag time to the start of the conversion reaction, qRT-QuIC enables an alternative quantification of prion infectivity in samples. The concentration of PrP^Sc^ can be calculated and quantified with much lower repeats using a calibration curve by seeding known amounts of reference PrP into qRT-QuIC [172,173]. qRT-QuIC assay, which uses a lag time, is more suitable than SD_50_ measurements for quantitative prion detection and high-throughput testing.

The RT-QuIC method is the fastest, easiest, least expensive, most practical, and most suitable cell-free conversion test for high-throughput testing. The RT-QuIC assay detected PrP^Sc^ at levels ranging from ~10^−15^ g to 10^−13^ g in brain homogenate, CSF, and nasal fluids from various animal species with high specificity [160]. QuIC has also been able to detect prions in samples such as blood [174,175], urine [176], saliva [177], feces [178], spinal cord, tonsils, lymph nodes [179], and skin [180]. The sensitivity of the RT-QuIC is similar to the in vivo bioassay in hamsters but is 50–200 times faster and much less expensive. RT-QuIC assay is also less labor-intensive and time-consuming than PMCA tests, which require sonication rather than shaking and the Western blot technique rather than fluorescence-based readouts. In addition, RT-QuIC is much more suitable for high-throughput testing than PMCA because it relies on simple, real-time, automated, and fluorescence-based readouts. 

The RT-QuIC assay allows rapid and highly sensitive differentiation of prion-infected animals from healthy animals. Furthermore, the RT-QuIC assay has been adapted to detect all three types of BSE (C-BSE, L-BSE, and H-BSE) and allows discrimination of these BSE types using specific rPrP^Sc^ substrates [161,181,182,183]. Using chimeric hamster-sheep substrate, RT-QuIC could detect C- and L-BSE-associated seeding activity in less than 48 h. The test was at least 10^4^-fold more sensitive than the IDEXX HerdCheck BSE-scrapie short assay and as sensitive as the infectivity bioassay [8]. The observation that hamster 23–231, human 23–231, and hamster 90–231 rPrP substrates selectively allowed detection of L-BSE but not C-BSE can be used for discriminating those two forms of BSE [181]. 

L-BSE, H-BSE, and C-BSE can be detected and differentiated on the basis of relative reactivities and lag phases obtained using the bank vole rPrP 23–230 and sheep rPrP ARR 25–234 substrates. The use of bank vole rPrP as substrate in RT-QuIC allowed the amplification of all three types of BSE (H-, L-, and C-BSE). However, if the same sample dilution was tested simultaneously with the sheep rPrP ARR 25–234 substrate, the lag phase is markedly longer for H-BSE, shorter for L-BSE, and undetectable for C-BSE. Thus, in practice, the test sample should be run simultaneously with the bank vole rPrP 23–230 and sheep Sh rPrP ARR 25–234 substrates in the same plate, enabling detection and discrimination of the strain in a single test. When brain samples contain a sufficient concentration of seeds, the method was at least as sensitive as bioassay and almost as sensitive as PMCA [161]. An improved version of RT-QuIC, also known as second-generation RT-QuIC, has been used to test CSF from goats infected with C-BSE and L-BSE. The reaction conditions of the second-generation RT-QuIC have been finely tuned to reduce processing time and achieve better sensitivity and specificity. This was possible using antibody 15B3-based immunoprecipitation and truncated hamster (residues 90–231) rPrP^Sen^ as the substrate, which produced a shorter lag phase than the full-length hamster rPrP (residues 23–231). Using this substrate, RT-QuIC was able to amplify undetectable quantities of PrP^Sc^ from CSF from goats in preclinical and clinical stages in less than 24 h [184]. 

This technique is revolutionizing the diagnostics of TSE. The quantitative aspect of RT-QuIC suggests that it can be reliable tool for evaluating anti-prion therapy. However, in contrast to PMCA, the fibrils produced in RT-QuIC do not mimic the pathologically infectious nature of the original seeds, limiting their applications in prion research studies. Because of the extremely high sensitivity of RT-QuIC, care must be taken to ensure the risk of contamination is minimized.

Trieschmann et al. developed a highly sensitive method base on seed-dependent PrP fibril formation that shows promising results in differentiating BSE-positive serum samples from healthy controls. The method is based on kinetic differences between seeded and unseeded amyloid β-protein aggregates of prion protein. In the first stage, synthetic prion protein is fluorescently labeled with a probe. Recombinant FITC-labeled bovine prion protein is then incubated at 37 °C for 20 h with continuous shaking with serum or plasma from infected cattle, and the samples are analyzed by flow cytometry. PrP^Sc^ aggregates present in the serum act as seeds and facilitate the formation of new, easily detectable amounts of labeled PrP aggregates, while, in the absence of seeds, PrP aggregate formation is inhibited. This method may provide the basis for an antemortem diagnostic test for BSE disease, which can detect extremely low amounts of prion protein aggregates in blood [185]. 

## 13. Conclusions

In the absence of effective treatments or vaccines, the early, rapid, and accurate diagnosis of BSE is crucial to prevent and control the spread of the disease. Therefore, it is very important to develop and select reliable and appropriate diagnostic tests. Many approaches have been explored, and methods implemented to improve BSE detection. Each test has its own unique strengths and drawbacks (Table 3), so several methods are combined to avoid the disadvantages of using a single method. Laboratories should use tests that have been fully evaluated and validated by the relevant regulatory authorities [186]. The choice of diagnostic methods depends on the purpose of the testing: screening, confirming, or strain typing. For screening, commercial rapid BSE tests are available for active disease surveillance in ELISA, Western blot, and immunochromatographic test formats. Confirmation of a positive BSE diagnosed by a rapid test requires the use of IHC for detection of the protein in formalin-fixed tissues or Western blot of tissue extracts where blots show characteristic patterns of protease-resistant protein PrP^res^. A positive diagnosis of BSE can only be made based on histopathology when the characteristic vacuolar changes in the brain with the typical neuroanatomic distribution are identified. Nevertheless, it is good practice to detect and confirm disease by a combination of at least two testing methods. However, it is worth noting that discrepancies in results may be due to differences in protocols at different laboratories, especially for in-house developed methods. In case of discrepancies between screening and confirmatory test results, further tests using immunochemistry or detection of characteristic fibrils by electron microscopy should be applied. The major obstacle to effective prevention and control of BSE is the lack of a test capable of identifying asymptomatic BSE-infected animals that could be used to screen seemingly healthy populations such as whole herds or specific animals for import or export purposes. In vitro prion techniques, including PMCA and RT-QuIC, were expected to represent a breakthrough in detecting minute amounts of prions in various tissues and body fluids, allowing for the first time to develop systems for antemortem TSE diagnosis. However, these tests have not been evaluated for use in legal BSE surveillance programs. 

## Figures and Tables

**Table 1:** The main characteristic features of BSE types.

	Classical BSE (C-Type)	Atypical BSE (H-Type and L-Type/BASE)
Source of infection	through an animal’s consumption of prion-contaminated feed	occur spontaneously at a very low rate in all cattle populations, does not appear to be associated with contaminated feed
Clinical signs	changes in behavior and temperament, hyperreactivity, hind-limb incoordination, weakness, and loss of body condition	some of the clinical signs of the disease include dullness, low head carriage, inactivity, or lack of nervousness
Transmission	efficiently transmitted orally to other species (e.g., sheep, goats, macaques)	low risk for oral transmission
Zoonotic potential	can be transmitted to humans through the consumption of contaminated meat causing variant Creutzfeldt–Jakob disease	transmission to humans has never been reported; the full risks presented to humans remain unknown, but some data suggesting that L-type BSE may be zoonotic
First diagnosis	1986, Great Britain	2004, Italy and France
Occurrence	worldwide distribution, cases have been reported in more than 20 countries, the implementation of appropriate control measures resulted in its decline, can affect cattle of any age	very rare disease, worldwide distribution, even in countries where no classical BSE has been reported, is expected to occur in all cattle populations, regardless of the control measures in place, occurs particularly in cattle older than 8 years
World Organisation for Animal Health (WOAH)	its occurrence may be considered for the purpose of the official BSE risk status recognition by the WOAH	its occurrence is not considered for the purpose of the official BSE risk status recognition by the WOAH
Biochemical characteristics of PrP^Sc^	extremely resistant to degradation by proteinase digestion	less resistant to degradation by proteinase digestion, can be degraded by stringent proteinase K digestion
WB pattern	Three bands correspond to the diglycosylated (~28 kDa), monoglycosylated (~22 kDa), and unglycosylated (~18 kDa) forms	L-type form has a lower while H-type higher molecular mass of the unglycosylated PrP^Sc^ when compared with classical BSE

**Table 2:** Characteristics of the monoclonal and polyclonal antibodies used in the BSE diagnosis.

Antibody		Epitope		Type	Application	Reference
Region	Name	Amino-Acid Sequence	Position			
N-terminal region	SAF32	QPHGGGW ^a^	62–92 ^b^	Monoclonal	EIA, FC, IHC, WB	[43]
	B103	QGGTHGQWNKPSKPKTNMK	103–121 ^b^	Polyclonal	IHC	[44]
	P4	WGQGGSH	101–107 ^b^	Monoclonal	WB, ELISA, IHC, PET-blot	[45]
	BG4	SPGGNRYPP	46–54 ^c^	Monoclonal	IHC	[16]
	8G8	SQWNKPSK	100–107 ^c^	Monoclonal	WB, IHC	[46]
	R521	GQGGSHSQWNKPGGC	94–105 ^c^	Polyclonal	WB	[47]
	R505	CSQWNKPSKPKTN	100–111 ^c^	Polyclonal	WB	[47]
	12B2	WGQGG	101–105 ^b^	Monoclonal	IHC, WB, IFMA	[48]
Core/globularregion	SHa31	YEDRYYRE	156–163 ^b^	Monoclonal	IFMA, WB, IHC	[43]
	F89/160.1.5	SRPLIHFGSDYEDR	146–159 ^b^	Monoclonal	IHC, WB, ICC/IF, ELISA	[49]
	12F10	SDYEDRYYRE	154–163 ^b^	Monoclonal	IHC	[43]
	6H4	YEDRYYREN	156–164 ^b^	Monoclonal	WB, IHC,	[50,51]
	T1	LIHFGND	141–147 ^c^	Monoclonal	IHC	[52]
	F89	IHFG	142–145	Monoclonal	IHC,	[49]
	L42	YEDRYY	156–161 ^b^	Monoclonal	WB, IHC, ELISA	[48]
	2G11	YRENMY	153–158 ^c^	Monoclonal	IHC	[53]
	132	AVVGGLGGY	119–127 ^d^	Monoclonal	ELISA, IFA	[54]
	9A2	WNK	110–112 ^b^	Monoclonal	WB, IFMA	[48]
C-terminal region	F99/97.6.1	QYQRES	228–233 ^b^	Monoclonal	IHC	[55]
	31C6	DWEDRYY	143–149 ^d^	Monoclonal	ELISA, IHC	[54]
	R145	YQRESQAYYQRGA	221–233 ^b^	Monoclonal	IHC, PET-blot	[48,56]
	44B1		155–231	Monoclonal	IHC, ELISA	[54]
	SAF84	RPVDQY	175–180 ^b^	Monoclonal	IHC, WB	[43,56]
	94B4	HTVTTTTK	198–205 ^b^	Monoclonal	WB, IFMA	[57]
	6H10	NA	NA	Monoclonal	IHC	[58]

^a^ epitope of octarepeat (partially); occurs as 5 respectively 6 successive sequences; ^b^ position of the antibody epitope(s) in the bovine protein sequence; ^c^ position of the antibody epitope(s) in the ovine protein sequence; ^d^ position of the antibody epitope(s) in the mouse protein sequence; NA—not available.

**Table 3:** The advantages and disadvantages of main methods for BSE diagnostic purposes.

Method	Advantages	Disadvantages
HA *	it is one of the least expensive morphological methods; preserves tissue morphology; paraffin-embedded and frozen tissue samples can be stored and accessed when required; it does not require any special equipment, results can be viewed using a conventional bright-field microscope	particular areas of the brain are needed; inaccurate hemisectioning could result in the complete loss of a target area for testing; multi-step procedure; tissue is highly processed and may lead to loss of information; subjective interpretation of results; requires good sample preservation; has lower sensitivity than other methods; time-consuming and laborious; requires specialized reagents and qualified personnel; less specific than IHC; low throughput; qualitative method
IHC *	high specific and sensitive; fresh, frozen, and autolyzed samples can be used; paraffin-embedded and frozen tissue samples can be stored and accessed when required; allows cellular localization of protein; relatively inexpensive; widely used; it does not require any special equipment, results can be viewed using a conventional bright-field microscope; verify the results obtained by HA	semi-quantitative; multi-step procedure; variability depends on the fixation procedure, staining protocol, and antibody selection; particular areas of the brain are needed; requires specialized reagents and qualified personnel; subjective interpretation of results; low throughput; non-specific reactions can occur, time-consuming and laborious
PET-blot	highly sensitive and specific; can be used to discriminate TSE strains, macroscopic observation possible	not a rapid tool; not suitable for large-scale screening; time-consuming and laborious; requires paraffin-embedded tissues, specialized equipment, reagents, and qualified personnel; has lower microscopic resolution than IHC; multi-step procedure; it is not commonly used; lack of standardization
WB *	very sensitive and specific; fresh, frozen, and autolyzed samples can be used; widely used; provide valuable information about the biochemical properties of PrP^Sc^; can be used to discriminate TSE strains; can be quantitative or qualitative; similar sensitivity to IHC	give no information on the neuroanatomical location of PrP^Sc^; requires specialized equipment, reagents, and qualified personnel; multi-step procedure; non-specific reactions can occur; subjective interpretation of results; paraffin-embedded tissues cannot be used; sensitivity varies between methods and laboratories
Rapid tests *	highly sensitive and specific; rapid; suitable for large-scale screening; full automation is possible; based on WB, immunochromatography, and ELISA; does not need ultracentrifugation steeps needed to concentrate the PrP^Sc^; can be quantitative or qualitative; fresh, frozen, and autolyzed samples can be used; can be used to discriminate TSE strains; most of them are commercially available	particular areas of the brain are needed; occasionally give false positive results; require specialized reagents and qualified personnel; some tests require specialized equipment; paraffin-embedded tissues cannot be used
IFA	cells or frozen tissue can be used; highly sensitive and specific; rapid,	expensive; requires specialized equipment (fluorescence or laser microscope), reagents, and qualified personnel; lack of standardization; frozen sections have poor morphology; non-specific results can occur; low throughput,
IFMA	extremely sensitive; allow simultaneous detection of multiple analytes; amenable to high-volume testing; can be used to discriminate TSE strains; automated	expensive; requires specialized equipment (cytometer), reagents, and qualified personnel; non-specific results can occur; rarely used; lack of standardization
SAF	highly specific; autolyzed sample can be used	results depend on the region used and sample purification scheme; labor-intensive; requires specialized equipment (electron microscope), reagents, and qualified personnel; less sensitive than WB; rarely used; only qualitative
Bioassay	the most sensitive and specific; provide information about PrP^Sc^ infectivity; strain-typing method	labor-intensive and time-consuming; not suitable for large-scale screening; have the highest sensitivity only when performed in homologous species
PMCA	extremely sensitive, can be used to understand the biology of prion proteins, can be quantitative or qualitative; can be automated; can be used for antemortem TSE diagnosis	requires specialized equipment (sonicator), reagents, and qualified personnel; no real-time detection; time-consuming; false-positive results may occur; lack of standardization; requires additional substrate purification step
RT-QuIC	extremely sensitivity, fast; most straightforward; practicable; relatively inexpensive; less labor-intensive and time-consuming than PMCA; automated; spontaneous fibrilization is minimized, real-time detection; can be quantitative or qualitative; can be used to discriminate TSE strains; suitable for large-scale screening; can be used for antemortem TSE diagnosis	no infectivity propagation; lack of standardization

* tests that should be used for confirmatory diagnosis of BSE according to Regulation (EC) No 999/2001 of the European Parliament and Council.

## Data Availability

Not applicable.
